# Behavior change interventions and policies influencing primary healthcare professionals’ practice—an overview of reviews

**DOI:** 10.1186/s13012-016-0538-8

**Published:** 2017-01-05

**Authors:** Bhupendrasinh F. Chauhan, Maya Jeyaraman, Amrinder Singh Mann, Justin Lys, Becky Skidmore, Kathryn M. Sibley, Ahmed Abou-Setta, Ryan Zarychanksi

**Affiliations:** 1 College of Pharmacy, University of Manitoba, Winnipeg, Canada; 2 Children’s Hospital Research Institute of Manitoba, Winnipeg, Canada; 3 George & Fay Yee Centre for Healthcare Innovation, Winnipeg, MB Canada; 4 Information Specialist Consultant, Ottawa, Canada; 5 Community Health Sciences, University of Manitoba, Winnipeg, Canada; 6 Department of Haematology and Medical Oncology, CancerCare Manitoba, Winnipeg, Canada; 7 Department of Internal Medicine, University of Manitoba, Winnipeg, Canada

## Abstract

**Background:**

There is a plethora of interventions and policies aimed at changing practice habits of primary healthcare professionals, but it is unclear which are the most appropriate, sustainable, and effective. We aimed to evaluate the evidence on behavior change interventions and policies directed at healthcare professionals working in primary healthcare centers.

**Methods:**

Study design: overview of reviews.

Data source: MEDLINE (Ovid), Embase (Ovid), The Cochrane Library (Wiley), CINAHL (EbscoHost), and grey literature (January 2005 to July 2015).

Study selection: two reviewers independently, and in duplicate, identified systematic reviews, overviews of reviews, scoping reviews, rapid reviews, and relevant health technology reports published in full-text in the English language.

Data extraction and synthesis: two reviewers extracted data pertaining to the types of reviews, study designs, number of studies, demographics of the professionals enrolled, interventions, outcomes, and authors’ conclusions for the included studies. We evaluated the methodological quality of the included studies using the AMSTAR scale. For the comparative evaluation, we classified interventions according to the behavior change wheel (Michie et al.).

**Results:**

Of 2771 citations retrieved, we included 138 reviews representing 3502 individual studies. The majority of systematic reviews (91%) investigated behavior and practice changes among family physicians. Interactive and multifaceted continuous medical education programs, training with audit and feedback, and clinical decision support systems were found to be beneficial in improving knowledge, optimizing screening rate and prescriptions, enhancing patient outcomes, and reducing adverse events. Collaborative team-based policies involving primarily family physicians, nurses, and pharmacists were found to be most effective. Available evidence on environmental restructuring and modeling was found to be effective in improving collaboration and adherence to treatment guidelines. Limited evidence on nurse-led care approaches were found to be as effective as general practitioners in patient satisfaction in settings like asthma, cardiovascular, and diabetes clinics, although this needs further evaluation. Evidence does not support the use of financial incentives to family physicians, especially for long-term behavior change.

**Conclusions:**

Behavior change interventions including education, training, and enablement in the context of collaborative team-based approaches are effective to change practice of primary healthcare professionals. Environmental restructuring approaches including nurse-led care and modeling need further evaluation. Financial incentives to family physicians do not influence long-term practice change.

**Electronic supplementary material:**

The online version of this article (doi:10.1186/s13012-016-0538-8) contains supplementary material, which is available to authorized users.

## Introduction

Approximately one in six Canadians aged 20 years or older suffer from chronic diseases such as diabetes, cardiovascular diseases, chronic respiratory diseases, arthritis, osteoporosis, mental illness, and cancer [1]. Combining direct medical costs ($38.9 billion) and indirect productivity losses ($54.4 billion), the total economic burden of chronic illness exceeds Canadian $93 billion a year [2]. Despite this enormous expenditure, 12 to 15% of Canadians feel they receive inadequate chronic disease care [3, 4]. The major unmet needs include long waiting periods for medical services [5] and unavailability of essential services [4]. Compared with people in other developed nations, Canadians today are less satisfied with their access to and quality of care [6] and have worse health outcomes for several medical conditions [7]. The numbers of patients with chronic diseases and the existing gap in quality of care present a significant challenge for public health policy-makers [8, 9].

With the objective of closing gaps in quality of care and managing patients with chronic diseases, the implementation of patient-centred treatment has recently gained attention from policy-makers [10–12]. Patient-centered medical centres may become the future backbone of the Canadian healthcare system [13]. These teams may include family physicians, physician assistants, nurses, pharmacists, social workers, mental health counselors/psychologists, dieticians, and midwives among others. To achieve efficient and effective patient-centered medical homes, some changes in the way healthcare is delivered will be required. To do so, effective behavior change interventions and supporting policies are required [14, 15]. However, it is unclear which intervention(s) and policies are appropriate, sustainable, and sufficiently safe to support practice change and improve patient-relevant outcomes in primary healthcare settings. Despite extensive published literature including randomized controlled trials [16, 17], observational studies [18, 19], and systematic reviews [20–22], no recent comprehensive review classifying or evaluating the feasibility or effectiveness of interventions and policies in terms of patients’ and professionals’ outcomes exists. The objectives of this overview of reviews were to identify, classify, and critically appraise reviews evaluating behavior change interventions and policies influencing primary healthcare professionals working at primary healthcare centers.

## Methods

### Data sources and searches

The search strategy was developed and tested through an iterative process by an experienced medical information specialist in consultation with the review team. We searched MEDLINE (Ovid), Embase (Ovid), CINAHL (EbscoHost), and the Cochrane Library (Wiley). Strategies utilized a combination of controlled vocabulary (e.g., “Physicians", "Primary Care”, “Physician’s Practice Patterns”, “Quality Improvement”) and keywords (e.g., family practitioner, home clinic, policy adherence). Vocabulary and syntax were adjusted across databases. Results were restricted to the English language and the dates from January 2005 to July 2015 (Additional file 1). We used DistillerSR (Version 2, Evidence Partners Inc. ON, Canada) for study selection, data extraction, and project management.

### Study selection

We included (1) systematic reviews, overview of reviews, scoping reviews, rapid reviews, or health technology assessments that (2) evaluated behavior change interventions or policies on primary healthcare professionals (including general practitioners/family physicians, physician assistants, nurses, pharmacists, social workers, mental health counselors/psychologists, dieticians, and midwives) (3) working at primary healthcare settings (4) reporting any outcomes of primary healthcare professionals’ practice change, and (5) published in the English language as full-text articles. Primary healthcare settings were defined as the provision of integrated, accessible health care services by clinicians who are accountable for addressing a large majority of personal health care needs, developing a sustained partnership with patients, and practicing in the context of family and community [23, 24]. Considering the application of outcomes in the Canadian context, reviews that exclusively included studies conducted in either underdeveloped or developing countries were excluded.

The abstracts and titles of relevant citations were independently screened by two reviewers to determine eligibility. The same two reviewers independently assessed the eligibility of full-text reports of relevant citations using a standardized pre-piloted form outlining the inclusion and exclusion criteria. Disagreements were resolved by consensus or with the involvement of a third reviewer, if needed.

### Data extraction and quality assessment

Two reviewers independently abstracted data from the included reviews using standardized piloted forms. The following data were extracted from each included review: review type, number and study designs that the review included, types of professionals evaluated, interventions, outcomes, therapeutic domains, and authors’ conclusions.

All behavior change interventions and policies were classified into nine categories of interventions and seven categories of policies following the behavior change wheel framework proposed by Michie et al*.* [15]. This framework consists of a behavior system at the hub, encircled by nine intervention functions and then by seven policy categories. The nine behavior change interventions include (1) education (increasing knowledge or understanding): e.g., continuous medical education; (2) persuasion (using communication to induce positive or negative feelings or stimulate action): e.g., reminders; (3) incentivization (creating expectation of reward): e.g., payment for performance; (4) coercion (creating expectation of punishment or cost): e.g., punishment or fines; (5) training (imparting skills): e.g., communication skills training; (6) restriction (using rules to reduce the opportunity to engage in the target behavior): e.g., rules for prohibiting the use; (7) environmental restructuring (changing the physical or social context): e.g., shared decision-making; (8) modeling (providing an example for people to aspire to or imitate): e.g., local opinion leaders; (9) enablement (increasing means/reducing barriers to increase capability or opportunity): e.g., clinical decision support systems. While the seven policies include: (1) communication/marketing (using print, electronic, telephonic or broadcast media): e.g., advertising media; (2) guidelines (creating documents that recommend or mandate practice): e.g., management guidelines; (3) fiscal (using the tax system to reduce or increase the financial cost): e.g., financial provisions from policy-makers; (4) regulation (establishing rules or principles of behavior or practice): e.g., rules and regulations; (5) legislation (making or changing laws): e.g., law amendments; (6) environmental/social planning (designing and/or controlling the physical or social environment): e.g., social support; (7) service provision (delivering a service): e.g., service or facilitation.

Two reviewers independently, and in duplicate, evaluated the methodological quality of the included reviews using the assessing the methodological quality of systematic reviews (AMSTAR) scoring system [25]. Conflicts were resolved by consensus or discussion with a third reviewer, if needed. Reviews with AMSTAR score ≥8, 4 to 7, ≤3 were considered as high, moderate, or low-methodological quality, respectively.

We summarized the findings that emerged from the subjective judgment matrix, which was based on the authors’ conclusions, qualitative data, quantitative data with statistically significant group differences in terms of patients’ and primary healthcare providers’ outcomes, and the methodological quality of included reviews [25–28]. The protocol for this overview of reviews has been developed prior to conduct the review and provided to the Primary Health Care Branch, Manitoba Health, Seniors and Active Living, Government of Manitoba, Canada. The Preferred Reporting Items for Systematic Reviews and Meta-Analyses (PRISMA) guidelines for reporting the systematic review were followed.

## Results

**Fig. 1:**
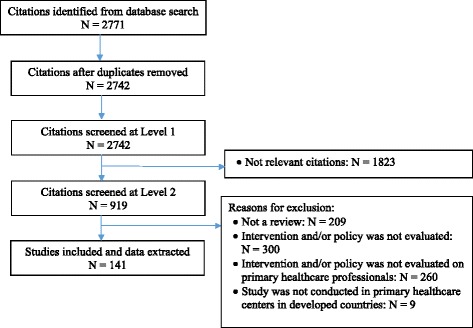
Flow diagram of the selection of citations

We screened 2771 citations and included 138 reviews representing 3502 individual studies (Fig. 1). The characteristics of the included reviews are presented in Table 1. Of the included studies, three were overviews of reviews [29–31]. Most reviews (91%) investigated behavior change interventions and policies among family physicians primarily managing chronic diseases at primary healthcare centers. We classified the included reviews into eight of nine categories of behavior change interventions including education (*n* = 28, 20%), enablement (*n* = 16, 12%), environmental restructuring (*n* = 18, 13%), incentivization (*n* = 7, 5%), modeling (*n* = 2, 2%), multiple interventions (*n* = 42, 30%), persuasion (*n* = 4, 3%), training (*n* = 11, 8%), and three of seven categories of policies including service provision (*n* = 5, 4%), communications (*n* = 3, 2%), and guidelines (*n* = 2, 2%). Major chronic diseases evaluated were mental disorders (*n* = 12, 9%), diabetes (*n* = 10, 7%), respiratory diseases (*n* = 8, 6%), cancer (*n* = 5, 4%), cardiovascular diseases (*n* = 4, 3%), arthritis/osteoporosis (*n* = 3, 2%), and hypertension (*n* = 2, 2%); some reviews reported more than one chronic disease. Total of 36 (26%) reviews exclusively included randomized controlled trials. The remaining reviews included systematic reviews, observational studies, interrupted time series studies, and controlled before-after studies (Table 1). Of the total included reviews, 68 (49%) reviews were of high quality, 60 (44%) reviews were of moderate quality, and 11 (8%) reviews were of low quality (Additional file 1: Table S1).

**Table 1:** Key features of included reviews

Study	Type of review	Study design included	Number of included studies	Professionals evaluated	Intervention(s)	Type of disease(s)	Funding
Behavior change interventions							
Education (increasing knowledge or understanding)							
Chhina et al. [32] 2013	SR	Any study design	15	FPs	Academic detailing	NR	No
Mostofian et al. [29] 2015	Overview	Reviews	14	FPs	Any interventions	NR	No
Velden et al. [33] 2012	SR	Any study design	58	FPs, others	Any interventions	RTIs	Yes
Thepwongsa et al. [20] 2014	SR	RCTs, non-RCTs, ITS	11	FPs	CME	NR	Yes
Thomas et al. [34] 2006	SR	Any study design	13	FPs	CME	NR	Yes
Ginige et al. [21] 2007	SR	Any study design	4	FPs	CME, video, text	Chlamydia	No
Brody et al. [35] 2013	SR	Any study design	16	FPs, nurses, SWs, pharmacists	Dementia educational/dissemination intention	Dementia	Yes
Schichtel et al. [36] 2013	SR	RCTs, cluster RCTs	21	FPs, Nurses, PAs	Education	Cancer	Yes
Hardy et al. [37] 2011	SR	Any study design	0	FPs	Education	Mental illness	No
Miller et al. [38] 2010	SR	Any study design	16	FPs	Education	NR	No
Lineker et al. [39] 2010	SR	Any study design	7	FPs, nurses	Education	Arthritis	No
Alvarez et al. [40] 2006	SR	Any study design	18	FPs	Education	Pallative care	No
Howe et al. [41] 2006	SR	RCTs	18	FPs	Education	NR	No
Kamarudin et al. [42] 2013	SR	Any study design	47	FPs	Education	NR	No
Thepwongsa et al. [43] 2014	SR	Any study design	13	FPs	Education	T2DM	Yes
Perry et al. [44] 2011	SR	Any study design	5	FPs	Educational meetings, audit-feedback, reminders, mass media, local opinion leaders	Dementia	Yes
Vodicka et al. [45] 2013	SR	Any study design	17	FPs, nurses	Educational or behavior change interventions	RTIs, otitis media	Yes
Guldberg et al. [46] 2009	SR	RCTs	10	FPs	Feedback	T2DM	Yes
Cheraghi-Sohi et al. [47] 2008	SR	RCTs	9	FPs	Feedback or training or both	NR	No
Ring et al. [48] 2007	SR	RCTs	14	FPs	Interactive educational seminar, QI learning collaborative for general practice teams	Asthma	Yes
Rourke et al. [49] 2015	MA	Any study design	37	FPs	Lecture, audit-feedback, computer based learing, multicomponent intervention	Skin lesions	No
Reinders et al. [50] 2011	SR	RCTs	10	FPs	Patient feedback	NR	Yes
Gijbels et al. [51] 2010	SR	Any study design	61	Nurses, midwives	Education	NR	Yes
Zaher et al, [52] 2012	SR	Any study design	13	FPs	Practice-based small group learning programs	NR	No
Curti et al. [53] 2015	SR, MA	RCTs, cluster-RCTs, CBA	12	FPs	Educational materials, meetings, CME, audit-feedbacks, reminders	Occupational diseases	No
Goulart et al. [54] 2011	SR	Any study design	20	FPs	Education	Skin cancer	Yes
Omidvari et al. [55] 2013	SR	RCTs	3	FPs	Guidelines	NR	Yes
Benthem et al. [56] 2009	SR	RCTs, CBA or ITS	27	FPs	Education	Psychiatric disorders	No
Enablement (increasing means/reducing barriers to increase capability or opportunity)							
Adaji et al. [57] 2008	SR	Any study design	29	FPs	Information technology	Diabetes	Yes
de Lusignan et al. [58] 2014	SR	Any study design	143	FPs	Access to electronic health records	NR	Yes
Pires et al. [59] 2014	SR	Any study design	18	FPs	Communication skills training for FPs	NR	No
Holstiege et al. [60] 2015	SR	RCTs; cluster RCTs	7	FPs	CDSSs	NR	No
Dixon et al. [61] 2013	SR	Any study design	10	FPs, others	Computer-based interventions	NR	Yes
Robertson et al. [62] 2010	SR	Any study design	21	Pharmacists	CDSSs	NR	Yes
Curtain et al. [63] 2014	SR	Any study design	8	Pharmacists	CDSSs	Allergic rhinitis, stroke	No
Souza et al. [64] 2011	SR	RCTs	41	FPs	CDSSs	Dyslipidaemia, cancer, mental illnesses	Yes
Fathima et al. [65] 2014	SR	RCTs	16	FPs, nurses, pharmacists, PAs	CDSSs	Asthma, COPD	No
Cleveringa et al. [66] 2013	SR	RCTs	20	FPs	CDSSs, feedback on performance	T2DM	Yes
Calabretto et al. [67] 2005	SR	RCTs	4	Pharmacists	Elecronic decision support system	NR	Yes
Boyle et al. [68] 2010	SR	Any study design	12	FPs	Electronic medical records	Tobacco dependence	Yes
Lainer et al. [69] 2013	SR	RCTs	10	FPs, pharmacists	Information technology	NR	Yes
Huang et al. [70] 2013	SR, MA	Any study design	13	FPs	Point of care testing	RTIs	No
Gialamas et al. [71] 2010	SR	RCTs, quasi-RCTs	6	FPs, others	Point of care testing	Diabetes, hyperlipedemia, coagulation disorders	Yes
Motulsky et al. [72] 2013	SR	Any study design	19	FPs, pharmacists	Second-generation electronic prescriptions	NR	No
Environmental restructuring *(changing the physical or social context)*							
Damiani et al. [73] 2013	SR	Any study design	26	FPs	Group versus single handed practice, information and communication technology	NR	No
Riley et al. [74] 2010	SR	Any study design	12	Others	Group visits	Diabetes	No
Unverzagt et al. [75] 2014	SR	RCTs	84	FPs	Multiple interventions	Cardiovascular	Yes
Gilbody et al. [76] 2008	MA	RCTs	16	FPs	Screening and case-finding instruments	Depression	Yes
Legare et al. [77] 2010	SR	Any study design	39	FPs, nurses, pharmacists, SWs, midwives	Shared decision-making	NR	Yes
Smith et al. [78] 2007	SR, MA	RCTs, CBA, ITS	20	FPs	Shared-care interventions	Chronic diseases	No
Mitchell et al. [79] 2008	SR	Any study design	18	FPs	Multidisciplinary primary care team	Stroke	Yes
Page et al. [80] 2005	SR	RCTs, non-RCTs, CBA	6	FPs, Nurses	Any interventions in nurese-led care	Coronary heart disease	No
Kuethe et al. [81] 2013	SR	RCTs	5	FPs, nurses, PAs	Nurse-led care	Asthma	No
Carey et al. [82] 2007	SR	RCTs	22	Nurses	Nurse-led care	Diabetes	Yes
Desborough et al. [83] 2012	SR	Any study design	13	FPs, Nurses	Nurse-led care	NR	Yes
Urquhart et al. [84] 2009	SR	RCTs, CBA, ITS	9	Nurses	Nursing record system	NR	Yes
Martelly et al. [85] 2014	SR, MA	RCTs	24	FPs, Nurses	Nurse-led care	NR	No
Laurant et al. [86] 2005	SR	RCTs, CBA, ITS	16	FPs, Nurses	Nurse-led care	NR	Yes
Courtenay et al. [87] 2008	SR	Any study design	21	Nurses	Nurse-led care	Pain	Yes
Dennis et al. [88] 2009	SR	Any study design	46	FPs, nurses, pharmacists	Task shifting	Chronic diseases	Yes
Health, [89] 2013	SR	RCTs, SRs	6	FPs, nurses	Task shifting	Chronic diseases	Yes
Proia et al. [90] 2014	SR	Any study design	80	FPs, nurses, pharmacists	Team based care	Blood pressure	No
Schadewaldt et al. [91] 2011	SR	RCTs	7	Nurses	Multiple interventions	Coronary artery disease	No
Incentivization (creating expectation of reward)							
Scott et al, [92] 2011	SR	RCTs, CBA, ITS	7	FPs	Financial incentives	NR	Yes
McDonald et al. [93] 2008	SR	Any study design	23	FPs	Funding initiatives or incentives	NR	Yes
Langdown et al. [94] 2014	SR	Any study design	11	FPs	P4P	Asthma, coronary heart disease, diabetes	No
Eijkenaar et al. [30] 2013	Overview	SRs	22	FPs	P4P	NR	No
Houle et al. [95] 2012	SR	Any study design	30	FPs	P4P	Chronic diseases	No
Gillam et al. [96] 2012	SR	Any study design	94	FPs	P4P	Chronic diseases	No
Vahidi et al. [97] 2013	SR	Any study design	11	FPs	Payment mechanisms to FPs	NR	Yes
Modeling (providing an example for people to aspire to or imitate)							
Flodgren et al. [98] 2011	SR	RCTs	18	FPs	Local opinion leaders	NR	Yes
Harkness et al. [99] 2009	SR, MA	RCTs, CBA, ITS	42	FPs, others	Mental health workers involvement	Mental health	Yes
Multiple interventions							
Zou et al. [115] 2012	SR	Any study design	8	FPs	Any interventions	STDs	Yes
Dwamena et al. [116] 2012	SR	RCTs, CBA, CCTs, ITS	43	FPs, nurses	Any interventions	General medical problems	Yes
Castelino et al. [117] 2009	SR	RCTs	12	Pharmacists	Interventions for prescribing	NR	No
Mansell et al. [118] 2011	SR	Any study design	22	FPs	Multiple interventions	Cancer	Yes
Guy et al. [119] 2011	SR	Any study design	16	FPs	Multiple interventions	Chlamydia screening	Yes
Laliberte et al. [120] 2011	SR, MA	Any study design	13	FPs, pharmacists	Multiple interventions	Osteoporosis	No
Jacobson et al. [121] 2011	SR	Any study design	15	FPs, nurses	Multiple interventions	Childhood obesity	No
Dennis et al. [122] 2008	SR	Any study design	164	FPs, nurses	Any interventions	NR	Yes
Grindrod et al. [31] 2006	Overview	SRs	34	Pharmacists	Any interventions	NR	No
Arnold et al. [123] 2005	SR	RCTs, quasi-RCT, CBA, ITS	39	FPs	Any interventions	NR	Yes
Moe-Byrne et al. [125] 2014 [124]	SR	SRs, studies	23	FPs	Any interventions	NR	Yes
McMillan et al. [125] 2013	SR	RCTs	30	FPs, nurses, others	Any interventions	NR	Yes
Loganathan et al. [126] 2011	SR	Any study design	16	FPs, nurses, Others	Any interventions	NR	Yes
Kaur et al. [127] 2009	SR	Any study design	24	FPs, pharmacists, others	Any interventions	NR	No
Okelo et al. [128] 2013	SR	Any study design	73	FPs, nurses, Pharmacists, others	Any interventions	Asthma	Yes
Huijg et al. [129] 2014	SR	Any study design	59	FPs, nurses, others	Any interventions	NR	Yes
Fahey et al. [130] 2005	SR	RCTs	72	FPs, nurses, pharmacists	Educational and organizational strategies	Hypertension	No
McKinstry et al. [131] 2006	SR	RCTs, quasi-RCTs, CBA, ITS	10	FPs	Informative, educational, multiple interventions	NR	No
Akbari et al. [132] 2008	SR	Any study design	17	FPs	Multiple interventions	NR	Yes
Gunten et al. [133] 2007	SR	Any study design	43	FPs, nurses, pharmacists	Pharmacists’ interventions	NR	No
Beach et al. [134] 2006	SR	RCTs	27	FPs	Provider and organization interventions	NR	No
Smit et al. [135] 2007	SR	RCTs	12	FPs, nurses, psychologists, others	Psychological and supportive interventions	Depression	No
Newhouse et al. [136] 2011	SR	Any study design	69	FPs, nurses, others	Advanced practice nurse care	NR	No
Lau et al. [137] 2012	SR, MA	Any study design	77	FPs, nurses	QI	Vaccination	Yes
Saxena et al. [138] 2007	SR	Any study design	9	FPs, nurses, others	Case management	Diabetes	No
Majka et al. [139] 2014	SR, MA	Any study design	15	FPs, nurses, dieticians, others	Care coordination and/or team approach methods; multiple simultaneous strategies	Patients with long term enteral tube feeding	No
Archer et al. [140] 2012	SR, MA	RCTs	79	FPs, nurses, pharmacists, psychologists	Colloborative care	Anxiety, depression	Yes
Thota et al. [141] 2012	SR, MA	RCTs	69	FPs	Collaborative care models	Depressive disorders	No
Christensen et al. [142] 2008	SR	RCTs, controlled trials	55	FPs, nurses, pharmacists, psychologists	Community models of care	NR	Yes
Phillips et al. [143] 2010	SR	Any study design	19	FPs	Different models using various interventions	NR	Yes
De Belvis et al. [144] 2009	SR	RCTs	13	FPs, nurses, PAs	Evidence based medicine tools	Diabetes	Yes
Sandall et al. [145] 2013	SR, MA	RCTs, cluster RCTs	13	FPs, midwives	Mid-wife led continuity model	NR	Yes
Baishnab et al. [146] 2012	SR	RCTs	3	FPs, Nurses	Organized asthma care	Asthma	Yes
Jackson et al. [147] 2013	SR	Any study design	19	FPs	PCMH	NR	Yes
Van Cleave et al. [148] 2012	SR	Any study design	23	FPs	QI initiatives, electronic records	NR	Yes
Shojania et al. [149] 2006	SR	RCTs, quasi-RCTs, CBA studies	58	FPs	QI strategies	T2DM	Yes
Tory et al. [150] 2015	SR	Any study design	7	FPs, pharmacists	QI measures	Osteoporosis	No
Gallagher et al. [151] 2010	SR	Any study design	9	Nurses, pharmacists	QI strategies	Hypertension, chronic kidney disease	Yes
Ranji et al. [152] 2008	SR	RCTs, CBA, ITS	43	FPs	QI strategies	NR	Yes
Gask et al. [153] 2011	SR	RCTs, CBA	13	FPs	Reattribution model	Medically unexplained symptoms	No
Rolfe et al. [154] 2014	SR	RCTs, quasi-RCTs, CBA	10	FPs	Interventions (informative, educational, behavioral, organizational)	NR	No
Persuasion (using communication to induce positive or negative feelings or stimulate action)							
Jenkins et al. [100] 2015	SR	Any study design	7	FPs	Audit-feedback, reminders, clinical decision support on imaging	Lower back pain	No
Holt et al. [101] 2012	SR, MA	CCTs	42	FPs	Reminders	NR	No
Siddiqui et al. [102] 2011	SR	RCTs	5	FPs	Reminders	Colorectal cancer screening	No
Lu et al. [103] 2008	SR	RCTs	164	FPs, pharmacists	Any interventions	Asthma, depression, Helicobacter pylori infection	Yes
Training (imparting skills)							
Moore et al. [104] 2013	SR, MA	RCTs, CBA	15	FPs, nurses, others	Communication skills training	Cancer	Yes
Eggenberger et al. [105] 2013	SR	RCTs, CCTs, CBA	12	FPs, nurses, SWs, psychologists, others	Communication skills training, education	Dementia	Yes
Horvat et al. [106] 2014	SR	RCTs, cluster RCTs, CCTs	5	FPs, nurses, PAs, psychologists, others	Cultural competence training	NR	No
Lie et al. [107] 2011	SR	Any study design	7	FPs, nurses, PAs	Cultural competency training	Blood pressure, diabetes	Yes
Henderson et al. [108] 2011	SR	RCTs, controlled studies	24	FPs	Cultural competency training	Chronic diseases	Yes
Soderlund et al. [109] 2011	SR	Any study design	10	FPs, nurses, PAs, SWs, psychologists, others	Motivational interviewing training	NR	Yes
Rashid et al. [110] 2010	SR	Any study design	8	Nurses	Nurse training	NR	No
Mesquita et al. [111] 2010	SR	Any study design	15	Pharmacists	Simulated patient methods	NR	Yes
Xu et al. [112] 2012	SR	Any study design	30	Pharmacists	Simulated-patient methods	Headache, abdominal pain	No
Sikorski et al. [113] 2012	SR, MA	RCTs	11	FPs	Training	Depression	Yes
Paskins et al. [114] 2014	SR	Any study design	28	FPs	Video stimulated recall	NR	Yes
Policy							
Service provision (delivering a service)							
OHTA [160] 2012	Report	SRs, MA, RCTs	7	FPs	Specialized community-based care	T2DM	Yes
Wilson et al. [156] 2006	SR	SRs, RCTs, CCTs, CBA	4	FPs	Any interventions altering consultation time	NR	Yes
McNaughton et al. [157] 2009	SR	RCTs	9	FPs	Brief non-pharmacological interventions	Depression	No
Wilson et al. [158] 2006	SR	RCTs, CCTs	7	FPs	Consultation time	NR	Yes
Bhanbhro et al. [159] 2011	SR	Any study design	17	FPs, nurses, pharmacists	Non-medical prescribing	NR	No
Communications (using print, electronic, telephonic or broadcast media)							
Jiwa et al. [161] 2014	SR	Any study design	18	FPs, others	Communications	NA	Yes
Cant et al. [162] 2011	SR	Any study design	20	FPs, dieticians	Dietitians’ correspondence practices	NR	No
Sawmynaden et al. [163] 2012	SR, MA	RCTs, quasi-RCTs, CBA, ITS	6	FPs	Email communication	NR	Yes
Guidelines (creating documents that recommend or mandate practice)							
Ramsaroop et al. [164] 2007	SR	Any study design	18	FPs	Advance Directive	NR	Yes
Clarke et al. [165] 2010	SR	Any study design	24	FPs	Guidelines	NR	Yes

*BP* blood pressure; *CBA* controlled before-after sudy; *CCTs* controlled clinical trails; *CME* continuing medical education; *COPD* chronic obstructive pulmonary disease; *FP* family physician; *ITS* interrupted time series study; *MA* meta-analysis; *NA* not applicable; *OR* odds ratio; *PAs* physician assistants; *P4P* pay-for-performance; *PCMH* patient-centered medical home; *PCPs* primary care providers; *RCTs* randomized clinical trails; *RD* risk difference; *RTIs* respiratory tract infections ; *SMD* standardized mean difference; *STD* sexually transmitted disease; *SR* systematic review; *SWs* social workers; *T2DM* type 2 diabetes mellitus; *WMD* weighted mean difference

### Behavior change interventions (Additional file 1: Table S1)

#### Education (increasing knowledge/understanding)

Twenty-eight reviews [20, 21, 29, 32–56] (*n* = 509 studies) evaluated educational interventions. Evidence from moderate- to high-quality reviews demonstrated that education to improve knowledge and skills [37–42, 48, 49, 51–56], continuing medical education [20, 21, 29, 34, 43], and academic detailing [32] were found to be effective in professional development to increase knowledge, optimize prescriptions, screening rate, and improve patient outcomes [20, 29, 32–36, 41, 44, 45, 50, 54]. Certain education interventions were evaluated as components of multifaceted education interventions, including interactive educational methods, reminder systems, audit and feedback, academic detailing, computer-based learning, lecture, as well as pamphlet in several reviews [29, 33, 36, 43, 44, 49]; which reported improvement in implementing guidelines into general practice [29], improved antibiotic prescribing [33], improved detection of cancer, dementia, and skin lesions [36, 44, 49]. Conflicting evidence exists on patient feedback. One review [50], based on ten studies, reported some evidence for the effectiveness of using feedback from real patients to improve knowledge and primary healthcare professionals’ practice change exists while other reviews [34, 46, 47] failed to reach the same conclusion.

#### Enablement (increasing means/reducing barriers to increase capability or opportunity)

Sixteen reviews [57–72] (*n* = 377 studies) evaluated the use of information technologies including interactive analysis systems [57–59, 69], clinical decision support systems [60, 62–66], electronic health records and prescriptions [61, 68, 72], and point of care testing [67, 70, 71] to increase capability and facilitate practice change of primary healthcare professionals. Evidence from moderate- to high-quality reviews demonstrated that enablement interventions improved communication between healthcare professionals and patients [59, 63], augmented knowledge [61], facilitated the appropriate antibiotic prescriptions [60], increased quality of service, reduced potential adverse events (drug interactions, contraindications, dose monitoring, and adjustment) [62], and improved several patient outcomes [64].

#### Environmental restructuring (changing the physical or social context)

Nineteen [73–91] (*n* = 470 studies) evaluated the impact of environmental restructuring including the use of collaborative or shared care practices or the institution of specialized nurses or other allied healthcare professionals [73, 74, 77–83, 85–91], or guideline implementation [75, 76] in primary healthcare settings. Evidence from poor- to high-quality reviews indicate organizational changes to increase collaboration among pharmacists, nurses, prevention coordinators, and other primary healthcare professionals led to increased physicians’ adherence to guidelines [75]. Nurse-led care was found to be as equally effective as general practitioners in patient satisfaction, asthma, cardiovascular, and diabetes management. However, weak study designs and restricted interventional scopes mean that further evaluation is required [80–82, 84], especially in the context of other chronic diseases.

#### Incentivization (creating an expectation of reward)

Seven reviews [30, 92–97] (*n* = 198 studies) evaluated the impact of financial incentives on family physicians. All reviews [30, 92–97] of poor- to high-quality failed to provide supportive evidence of any significant improvement in family physicians’ behavior change. One high-quality review [96] observed modest improvements in quality of care for chronic diseases, albeit, the impact on costs, professional behavior, and patient experience remained uncertain.

#### Modeling (providing an example for people to aspire or imitate)

Two reviews [98, 99] (*n* = 60 studies) evaluated modeling using local opinion leaders [98], or mental health workers [99] in primary healthcare settings. Evidence from moderate- to high-quality reviews demonstrated that involving local opinion leaders or subject experts to promote evidence-informed practices decreased the rates of consultations and prescriptions [98, 99].

#### Persuasion (using communication to induce positive or negative feelings or stimulate action)

Four reviews [100–103] (*n* = 218 studies) reported on interventions categorized as persuasion. Evidence from moderate- to high-quality reviews indicates that reminders [100–103] worked well to reduce unnecessary imaging for lower back pain [100] while improving the rate of screening [101] and vaccination [101].

#### Training (imparting skills)

Eleven reviews [104–114] (*n* = 165 studies) focused on training. Evidence from moderate- to high-quality reviews [104–114] reported that training on communication skills and cultural competency improved knowledge and professional expertise, which resulted in improved clinical outcomes including quality of life, well-being of patients with dementia, and reduced chronic disease in culturally and linguistically diverse communities [104–106, 108, 109, 113, 114].

#### Multiple interventions

Several reviews were focused on how to better manage chronic diseases using any behavior change interventions. To avoid misclassification, we classified these reviews under an umbrella term, multiple interventions. Forty-one reviews [31, 115–154] (*n* = 1375 studies) of poor- to high-quality focused on multiple interventions. The use of computer alerts within electronic medical records increased screening for sexually transmitted diseases [115]. Interventions in pharmacy services reduced suboptimal prescribing [117, 127, 133], and educational interventions improved primary healthcare providers’ identification, assessment, prevention and/or management of obesity in children and adolescents to achieve weight loss [121]. No review focused exclusively on audit and feedback, but multifaceted audit/feedback, reminders, educational outreach visits, and patient-mediated interventions [31, 116, 118, 119] were found to be effective in influencing health professionals’ prescribing practice. Financial incentives combined with educational interventions and audit/feedback have been found to be effective in increasing the practice of generic prescribing [124]. Multifaceted interventions where educational interventions occurred at many levels may be successfully incorporated into established medical communities after addressing local barriers to change [120, 123, 130, 153]. Advance practice nurse care [136], quality improvement strategies [137, 148–152], case management [138], collaborative care [140], evidence-based medicine practice strategies [144], midwife-led continuity services [145], comprehensive asthma care [146], and patient-centered medical home [125, 147] have all been evaluated. Moderate- to high-quality reviews demonstrated improved safety, quality care, increased vaccination rate, and improved management of patient with depression and anxiety in primary healthcare settings [135–137, 139–142, 144, 147, 148, 150, 151]. Few reviews failed to provide any conclusive outcomes [122, 126, 129, 131, 134, 143, 154, 155].

### Policies (Additional file 1: Table S1)

#### Service provision (delivering a service)

Five reviews [156–160] (*n* = 44 studies) of poor- to high-quality evaluated effects of consultation time [156, 158], brief non-pharmacological interventions (computer-based cognitive-behavioral therapy) [157], and non-medical prescribing [159] (drug prescriptions by nurses, pharmacists, and allied health professionals) on behavioral change of primary healthcare professionals. While a health technology report [160] assessed evidence on specialized community-based care and concluded that specialized community-based care effectively improves outcomes in patients with heart failure, chronic obstructive pulmonary disease, and diabetes. Bibliotherapy, cognitive behavioral therapy-based websites, and cognitive behavioral therapy-based computer programs [157] found to be effective in improving management of patients with depression. Other reviews [156, 158, 159] were not found to be effective.

#### Communication (using print, electronic, telephone, or broadcast media)

Three reviews [161–163] (*n* = 44 studies) of moderate- to high-quality evaluated communication as an intervention reporting inconclusive results. One review [161] uniquely assessed whether patients benefit from improved communication between primary healthcare practitioners and nephrologists. The review found little evidence of benefit from enhancing the quality of letters from specialists to primary healthcare practitioners.

#### Guidelines (creating documents that recommend practice standards)

Two reviews [164, 165] (*n* = 42 studies) of moderate- to high-quality evaluated the impact of guidelines on the improvement of healthcare professionals’ practice. None of the interventions found to be effective method for increasing advance directive completion rates in the primary healthcare setting [164, 165].

## Discussion

In our overview of reviews, we identified, classified, and evaluated the behavior change interventions and policies influencing practice change of primary healthcare professionals who primarily manage patients with chronic diseases at primary healthcare centers. Interactive and multifaceted continuous medical education programs including training with audit and feedback, and clinical decision support systems were found to be of benefit in improving knowledge, optimizing prescriptions, increasing screening rate, enhancing patient outcomes, and reducing adverse events. Limited evidence on environmental restructuring and modeling were found to be effective in improving collaboration and adherence to treatment guidelines. Collaborative team-based approaches involving primarily family physicians, nurses, and pharmacists were found to be effective. Limited evidence on nurse-led care approaches were found to be promising and warrant further evaluation using better study designs for different chronic diseases. Evidence clearly does not support the use of financial incentives to family physicians, especially for long-term sustained behavior and practice change.

To the best of our knowledge, so far this is the largest comprehensive overview of reviews evaluating authors’ reported efficacy of behavior change interventions and policies influencing primary healthcare professionals’ practice change and classified according to the behavior change wheel proposed by Michie et al*.* [15]. Our outcomes support the inferences reported by other overview reviews [166] and review [167] focused on individual interventions. Grimshaw and colleagues [166] reported that educational outreach (for prescribing) and reminders were found to be most promising approaches. Multifaceted interventions targeting different barriers to change are more likely to be effective than single interventions. We reported that education intervention found to be effective, especially when used as multifaceted interventions to achieve primary healthcare professionals’ practice change to improve quality of care and better manage patients with chronic diseases. Ivers and colleagues [167] reported audit and feedback generally leads to small but potentially important improvements in professional practice. We did not find any review exclusively evaluating audit and feedback on primary healthcare professionals; however, it was used with other interventions (e.g., education and training) and provided mixed results. With regards to financial incentives, Flodgren and colleagues have reported that financial incentives may be effective in changing healthcare professional practice [168]. In contrast, we found that financial incentives were not effective in practice change of family physicians working at primary healthcare centers.

This review did identify limited evidence on a few promising interventions, including nurse-led approaches and use of opinion leaders or specialists. Further, thorough evaluation in specific areas of interest should be performed before they are widely implemented in a healthcare setting.

To reduce the gap in quality of care and better manage patients with chronic diseases, behavioral interventions and supporting policies are essential. Through this overview of reviews, we attempted to provide an evidence to improve our understanding on which behavioral interventions and policies are effective to influence practice of primary healthcare professionals working in primary health care settings. This review is heavily weighted by evidence on family physicians, thus indicating the need for studies on other primary healthcare professionals. We excluded reviews that either evaluated these interventions and policies on specialists and hospital settings or included studies conducted exclusively in low- to middle-income countries, where the functionality of healthcare systems is different than Canada. Behavior change interventions or policies were classified based on the framework proposed by Michie and colleagues [15] and no other frameworks were explored or compared. Considering this is an overview of reviews and we have not performed a meta-analysis, we did not attempt to review individual studies from included reviews; there is a possibility of few studies might have been included by multiple reviews or might be a chance of over representation of outcomes. Evidence ranged from poor- to high-quality as well the high heterogeneity in interventions, study population, and outcomes prevented to generalize the conclusion to specific category of primary healthcare professionals or interventions and policies.

## Conclusion

Behavior change interventions including interactive and multifaceted continuous medical education, training with audit and feedback, enablement through advanced information technology-based systems, and collaborative team-based interventions can effectively modify healthcare professionals’ practice and patient outcomes. Limited evidence exists to support environment restructuring and modeling. Nurse-led systems of care warrant further evaluation. Financial incentives to family physicians do not influence long-term behavior and practice change.

## Additional file

Additional file 1: Table S1. Outcomes and methodological quality assessment of included reviews. (DOC 662 kb)
