# Erratum to: Behavior change interventions and policies influencing primary healthcare professionals’ practice—an overview of reviews

**DOI:** 10.1186/s13012-017-0568-x

**Published:** 2017-03-17

**Authors:** Bhupendrasinh F. Chauhan, Maya M. Jeyaraman, Amrinder Singh Mann, Justin Lys, Becky Skidmore, Kathryn M. Sibley, Ahmed M. Abou-Setta, Ryan Zarychanski

**Affiliations:** 1 College of Pharmacy, University of Manitoba, Winnipeg, Canada; 2 Children’s Hospital Research Institute of Manitoba, Winnipeg, Canada; 3 George & Fay Yee Centre for Healthcare Innovation, Winnipeg, MB Canada; 4 Information Specialist Consultant, Ottawa, Canada; 5 Community Health Sciences, University of Manitoba, Winnipeg, Canada; 6 Department of Haematology and Medical Oncology, CancerCare Manitoba, Winnipeg, Canada; 7 Department of Internal Medicine, University of Manitoba, Winnipeg, Canada

## Erratum

After publication of the original article [1] it was brought to our attention that authors Maya M. Jeyaraman, Ahmed M. Abou-Setta and Ryan Zarychanski were incorrectly included as Maya Jeyaraman, Ahmed Abou-Setta and Ryan Zarychanksi. The corrected names are included in the author list of this erratum.
