# Supplementary material for: Behavior change interventions and policies influencing primary healthcare professionals’ practice—an overview of reviews
Source: Implement Sci. 2017 Jan 5;12:3. doi: 10.1186/s13012-016-0538-8 (PMC5216570; doi:10.1186/s13012-016-0538-8)
Supplement: Additional file 1: Table S1. — Outcomes and methodological quality assessment of included reviews. (DOC 662 kb) [file 13012_2016_538_MOESM1_ESM.doc]

# Additional file 1: Table S1. Outcomes and methodological quality assessment of included reviews.

| **Study** | **Objective** | **Intervention** | **Outcomes** | **Main results** | **Authors’ conclusions** | **AMSTAR score** | **Effect** |
| --- | --- | --- | --- | --- | --- | --- | --- |
| **Behavioural change interventions**  **Education** *(Increasing knowledge or understanding)* | | | | | | | |
| Curti 2015 | To evaluate the effects of interventions to increase the reporting of occupational diseases by physicians | Educational materials, meetings, CME, audit-feedbacks, reminders | Rate of reporting of occupational disease | The use of educational materials with or without meetings did not considerably increase the rate of reporting when compared to no intervention. The use of an educational campaign increased the number of physicians reporting occupational diseases when compared to no intervention | Educational materials, educational meetings, or a combination of the two do not considerably increase the reporting of occupational diseases | 11 | **_** |
| Thepwongsa 2014 | To assess the effects of educational interventions targeted at general practitioners’ diabetes management | Education | GPs’ satisfaction, knowledge, behaviour changes, process of care and clinical outcomes | Fewer than half of the studies (5/13, 38.5%) reported a significant improvement in satisfaction with the programme, knowledge and practice behaviour | Paucity of studies; Evidence to support the effectiveness of education is partial and weak | 10 | **-** |
| Omidvari 2013 | To assess the effectiveness of nutritional screening in improving quality of care and patient outcomes compared with usual care | Guidelines | Process and patient outcomes | Physicians were receptive to the screening intervention, but the intervention did not result in any improvements in the malnutrition detection rate or nutritional intervention rate | Current evidence does not support the effectiveness of nutritional screening | 9 | **-** |
| Cheraghi-Sohi 2008 | To review the efficacy of (i) feedback of real patient assessments of interpersonal care skills, (ii) brief training focused on the improvement of interpersonal care (iii) interventions combining both (i) and (ii) | Feedback or training or both | Improvement in the interpersonal skills of primary care physicians | The only trial on training and one trial on feedback reported significant improvements | There is limited evidence concerning the effects of patient based feedback and no effect of training in the current status | 9 | **-** |
| Mostofian 2015 | To identify effective methods of implementing clinical research findings and clinical guidelines to change physician practice patterns, in surgical and general practice | Any interventions | Change physician practice patterns, in surgical and general practice | Active approaches, such as academic detailing, led to greater effects than traditional passive approaches. Positive change in physician behaviour was noted when exposed to active educational methods and multifaceted interventions | Active forms of CME and multifaceted interventions were found to be the most effective methods for implementing guidelines into general practice | 8 | **+** |
| Rourke 2015 | To assess the effectiveness of educational practices to improve the detection, categorization, and identification of skin lesions | Lecture, audit-feedback, computer based learing, multicomponent intervention | Improvement in the detection, categorization, and identification of skin lesions | 7 different educational practices evaluated; the pooled effect of the practices on participants’ abilities was large, with an SMD of 1.06 (95% CI, 0.81-1.31) | The most effective approache was to engage participants in a number of coordinated activities for an extended period. | 8 | **-** |
| Thepwongsa 2014 | To assess effects of online educational interventions targeted at general practitioners | CME | GP satisfaction, knowledge, behavioural changes, process of care, clinical outcomes | 72.7% studies found a significant improvement in outcomes | Online CME could improve GP satisfaction, knowledge and practices | 8 | **+** |
| Schichtel 2013 | To examine the effectiveness of educational interventions for PCPs to promote the early diagnosis of cancer | Education | Improvement in diagnosis of cancer | Interactive education, computerised reminder systems, and audit and feedback delivered to clinicians may significantly increase several cancer detection measures in the short term and some evidence that they promote early diagnosis | Certain educational interventions delivered at a clinician as well as at a practice level may promote the early diagnosis of cancer in primary care | 8 | **+** |
| Vodicka 2013 | To assess the effectiveness of primary care-based interventions to reduce antibiotic prescribing for children with RTIs | Educational or behaviour change interventions | Change in clinicians’ antibiotic prescribing for acute RTIs in children | Interventions that combined parent education with clinician behaviour change decreased antibiotic prescribing rates by between 6–21%. Automatic computerised prescribing prompts increased prescribing appropriateness | Interventions directed towards parents and/or clinicians can reduce rates of antibiotic prescribing | 8 | **+** |
| Perry 2011 | To determine the effects of educational interventions about dementia, directed at PCPs | Educational meetings, audit-feedback, reminders, mass media, local opinion leaders | PCPs’ knowledge and attitude | A small group workshop and a decision support system increased dementia detection rates. An interactive seminar raised GPs’ suspicion of dementia. Adherence to dementia guidelines only improved when an educational intervention was combined with the appointment of dementia care managers | Educational interventions for PCPs that require active participation improve detection of dementia. Educational interventions alone do not seem to increase adherence to dementia guidelines | 8 | **+** |
| Reinders 2011 | To assess the effect of patient feedback interventions as a method of improving physicians’ consultation | Patient Feedback | Change in actual performance or outcomes of consultation skills | Only 4 of the 7 studies that assessed change in actual performance or results. | Some evidence for the effectiveness of using feedback from real patients to improve knowledge and behaviour exists | 8 | **+** |
| Chhina 2013 | To assess the effectiveness and the magnitude of the effect, of academic detailing, at modifying drug prescription behaviour of FPs in primary care settings | Academic detailing | Change in prescription rate | Half (7 of 15) of the includes studies found effective on medication prescribing behaviour of FPs. The median difference in relative change among the studies reviewed was 21% (interquartile range 43.75%) for RCTs, and 9% (interquartile range 8.5%) for observational studies | Academic detailing can be effective at optimizing prescription of medications by FPs | 7 | **+** |
| Zaher 2012 | To identify the format, content, and effects of practice-based small group learning programs involving FPs | Practice-based small group learning programs | Learners’ perceptions and learning outcomes | Evaluations of learners’ perceptions and learning outcomes indicate that practice-based small group learning programs constitutes a feasible and effective method of professional development | Current evidence suggests that practice-based small group learning programs may be an effective method of continuing professional development for FPs | 7 | **-** |
| Goulart 2011 | To determine the extent of evaluated interventions designed to educate PCPs about skin cancer, including melanoma | Skin cancer education | Knowledge, competence, confidence, diagnostic performance, or systems outcomes | Most studies (18/20, 90%) found a significant improvement in at least one of the following five outcome categories: knowledge, competence, confidence, diagnostic performance, or systems outcomes | Only a few interventions have been tested rigorously or evaluated under sufficient standardized conditions to allow for quantitative comparison | **7** | **+** |
| Ring 2007 | To investigate how best to encourage health professionals to promote, and for people with asthma to use, asthma action plans | Interactive educational seminar, QI learning collaborative for general practice teams | Promotion of asthma action plans | Only 2 of 14 included studies applied interventions on primary care professionals | Inadequate data to provide any firm conclusion | 7 | **-** |
| Brody 2013 | To examine patient and provider outcomes related to interprofessional education in dementia | Dementia educational/ dissemination intention | Health professional knowledge or behavioural outcomes | Most studies showed improvement either in clinician knowledge, confidence, and attitudes, or patient outcomes, few were able to link between the two, or measured the effects of the intervention over a long period of time | Improvement in clinician knowledge, confidence, and attitudes or patient outcomes were noted | 6 | **+** |
| Gijbels 2010 | To evaluate the impact of post-registration nursing and midwifery education on practice | Education | Practice change | Attendees benefit from post-registration programmes in relation to changes in attitudes, perceptions, knowledge, and in skill acquisition | The impact of post-registration nursing and midwifery education on practice has yet to be fully explored through a more systematic and coherent programme evaluation approach | 6 | **-** |
| Benthem 2009 | Is evidence available for the effectiveness of specific educational methods to teach GP trainees psychiatric diagnostic skills? | Teaching of psychiatric diagnostics | Change in diagnostic competence | No conclusive evidence has been found for the effectivity of an isolated educational method | No firm conclusion was derived | 6 | **-** |
| Howe 2006 | To assess effectiveness of educational interventions in primary care mental health care | Education | Patient and physician outcomes | Key features of significant outcomes were multifaceted interventions including practice based components in the intervention such as practice visit, use of own patients cases | Multifaceted interventions are effective | 6 | **+** |
| Kamarudin 2013 | To review the educational interventions to improve prescribing and identify educational methods that improve prescribing competency in both medical and non-medical prescribers | Education | Improvements in prescribing competency or performance | CME in the form of academic detailing and personalised prescriber feedback yielded positive results | The WHO Guide to Good Prescribing has the largest body of evidence to support its use and is a model for the design of targeted prescribing courses | 5 | **-** |
| Velden 2012 | To assess the effectiveness of physician-targeted interventions aiming to improve antibiotic prescribing for RTIs in primary care | Any interventions | Improvement in use of antibiotics | There was a 60% significantly improved antibiotic prescribing; interventions aiming to decrease overall antibiotic prescription were more frequently effective than interventions aiming to increase first choice prescription | This review emphasises the importance of physician education in optimising antibiotic use | 5 | **+** |
| Lineker 2010 | To evaluate the influence of educational programs designed to implement clinical practice guidelines for osteoarthritis and rheumatoid arthritis in primary care | Education | Behavioural outcomes | Peer facilitated workshops with nurse case-management support for patients decreased the number of referrals to orthopedics by 23%, and educational outreach by trained physicians improved prescribing of analgesics | There was limited literature on educational programs for the implementation of arthritis clinical practice guidelines in the primary care environment | 5 | **-** |
| Guldberg 2009 | To assess the effect of feedback to GPs on the quality of care for people with T2DM | Feedback | Quality of care for T2DM | Clinical outcomes like lowering of BP, Hba1c and cholesterol levels were seen in few studies | Feedback for quality improvement in diabetes care needs further evidence, especially of electronic feedback | 5 | **-** |
| Ginige 2007 | To review the evidence for specific interventions aimed at increasing chlamydia screening rates in primary care | CME, video, text | Any difference in age- and sex-specific chlamydia screening rates | The primary intervention was educational package targeting primary care physicians. The intervention was associated with an increase in screening rates of between 100% and 276% (p < 0.04) | There are only a limited number of randomized or controlled studies that demonstrate improved chlamydia screening of younger women in primary care | 5 | **+** |
| Alvarez 2006 | A review of educational interventions in palliative care for PCPs was performed, to evaluate professional practice change | Education | Measures of treatment for symptom control, learning culture of physicians, communication | Educative methods were role model training, small group discussions, and distribution of guidelines. Opioids prescription improved in two studies. Knowledge improved in all studies. Some benefit of multifaceted approach was stated | Palliative care education for PCP is poorly studied | 5 | **-** |
| Thomas 2006 | To assess CME, Continuing Professional Development, and Knowledge Translation and its effects on improving care of older patients by GPs | CME | Improvement of older patients care | Studies of CME have shown that the most effective programs for knowledge translation in these circumstances involve what is known as active-mode learning, which relies on interactive, targeted, and multifaceted techniques. | Multiple educational efforts such as written materials or toolkits combined with feedback and strong communication channels between instructors and learners were the most effective methods to change behaviours | 5 | **+** |
| Hardy 2011 | To evaluate the current evidence of the intervention's efficacy of education on healthcare professionals in primary and secondary care to monitor the physical health of people with serious mental illness | Education | Health improvement in patients with serious mental illness | No study was eligible. | None study evaluated education as intervention on professionals to improve patients with serious ill mental health | 2 | - |
| Miller 2010 | To investigate workplace based assessment affects doctors’ education and performance | Education | Doctors’ education and performance | Half (8 of 16) studies examined multisource feedback with mixed results; most doctors felt that multisource feedback had educational value, although the evidence for practice change was conflicting | There are few published articles exploring its impact on doctors’ education and performance. There is no evidence that alternative workplace based assessment tools (mini-clinical evaluation exercise, direct observation of procedural skills, and case based | 2 | **-** |
| **Enablement** (Increasing means/reducing barriers to increase capability or opportunity) | | | | | | | |
| Huang 2013 | To examine the association between point of care testing C-reactive protein and antibiotic prescribing for RTIs in general practice | Point of care testing | Antibiotic prescribing | Point of care testing C-reactive protein was associated with a significant reduction in antibiotic prescribing at the index consultation (RR 0.75, 95% CI = 0.67 to 0.83), but was not associated with antibiotic prescribing at any time during the 28-day follow-up period (RR 0.85, 95% CI = 0.70 to 1.01) or with patient satisfaction (RR 1.07, 95% CI = 0.98 to 1.17) | Point of care testing C-reactive protein significantly reduced antibiotic prescribing at the index consultation for patients with RTIs | 9 | **+** |
| Souza 2011 | To assess the effects of computerized clinical decision systems for primary preventive care on process of care, patient outcomes, harms, and costs | CDSSs | Process of care, patient outcomes, harms, and costs | Computerized clinical decision systems improved the process of care in 25 of 40 (63%) RCTs. Strong evidence supports the effectiveness of computerized clinical decision systems for screening and management of dyslipidaemia in primary care | Evidence supports the effectiveness of computerized clinical decision systems for screening and treatment of dyslipidaemia in primary care | 9 | **+** |
| Holstiege 2015 | To assess the effectiveness of CDSSs in improving antibiotic prescribing in primary care | CDSSs | Improvement antibiotic prescribing behaviour | 5 out of 7 trials showed marginal to moderate statistically significant effects of CDSSs in improving antibiotic prescribing behaviour | CDSSs found to be effective in improving antibiotic prescribing behaviour in primary care | 8 | **+** |
| Fathima 2014 | To evaluate effectiveness of computerized clinical decision systems in the care of people with asthma and COPD | CDSSs | Improvement in health outcomes | The use of computerized clinical decision systems improved asthma and COPD care in 14 of the 19 studies reviewed (74%). Nine of the 19 studies showed statistically significant (p < 0.05) improvement in the primary outcomes measured | Evidence supports the effectiveness of computerized clinical decision systems in the care of people with asthma. There is considerable improvement in the health care process measures and clinical outcomes by using computerized clinical decision systems | 8 | **+** |
| Cleveringa 2013 | To assess computerized decision support to improve diabetes care in primary setting | CDSSs, feedback on performance | Clinical performance and/or patient outcome | Computerized decision support with reminders, feedback on performance, and case management improved both patient outcome and the process of care | Computerized decision support used by healthcare providers in primary T2DM care are effective in improving the process of care; adding feedback on performance and/ or case management may also improve patient outcome | 8 | **+** |
| Robertson 2010 | To assess the impact of CDSSs targeting pharmacists on physician prescribing, clinical and patient outcomes | CDSSs | Prescribing outcomes | CDSSs addressing safety issues were more effective than CDSSs focusing on quality wse of medicines (10/11 versus 4/10 studies reporting statistically significant improvements in favour of CDSSs on ≥50% of all outcomes reported; P = 0.01) | Greater effectiveness of safety-focused compared with quality use of medicine-focused CDSSs was noted | 8 | **+** |
| deLusignan 2014 | To assess the effect of providing patients online access to their electronic health record and linked transactional services on the provision, quality, and safety of healthcare | Access to electronic health records | Change in quality or safety | No studies reported changes in health outcomes; though eight detected medication errors and seven reported improved uptake of preventative care. Professional concerns over privacy were reported in 14 studies | Patient online access and services offer increased convenience and satisfaction. Studies correcting medication errors may improve patient safety | 7 | **-** |
| Dixon 2013 | To evaluate computer-based interventions aimed at improving bidirectional communication between clinical and public health | Computer-based interventions | Improvement in bidirectional communication between clinicians and public health | Nascent and need further research and practice related to alerting clinicians about emerging threats. | Increasingly available clinical information systems make it possible to deliver timely, relevant knowledge to frontline clinicians in support of population health | 7 | **-** |
| Lainer 2013 | To assess the effects of information technology interventions on medication safety in primary care | Information Technology | Medical safety | Computerized provider order entry with clinical decision support decreased the prescription of potentially inappropriate medication or unsafe prescribing in pregnancy | Information technology interventions with inter-professional communication appear to be effective | 7 | **+** |
| Gialamas 2010 | To assess the analytical performance, clinical effectiveness, cost, and satisfaction of patients and health professionals with point of care testing for monitoring patients with diabetes, with hyperlipidaemia or requiring anticoagulant | Point of care testing | Analytical performance, clinical effectiveness, cost, Patient satisfaction and acceptability, health professionals' satisfaction and acceptability | No study found a statistically significant difference between point of care testing and pathology laboratory testing | No robust evidence was found that point of care testing in general practice improves patient health outcomes, that it has comparable analytical quality to pathology laboratory testing, that it is cost-effective compared to usual care | 6 | **-** |
| Pires 2014 | To assess the communication between patients and health professionals with the application of the Roter Interaction Analysis System methodology | Communication skills training for FPs | Health outcomes | Communication using Roter Interaction Analysis System tool found effective | The communication between health professionals and patients using Roter Interaction Analysis System has produced concrete results | 5 | **+** |
| Motulsky 2013 | To describe second-generation electronic prescription technologies and identify their impacts on the medication management process in primary care | Second-generation electronic prescriptions | Medication management process in primary care | The only demonstrated impacts of second-generation technologies were found at two levels: positive impacts on the quality of the pharmacological profile available to professionals, and negative impacts on the execution of prescriptions in pharmacies | There is little empirical data demonstrating benefits to second-generation technologies, even if it is a highly promoted model for improving primary care quality | 5 | **-** |
| Boyle 2010 | To evaluate the use of electronic medical records to increase the use and impact of clinical guidelines to promote tobacco cessation treatment in primary care settings | Electronic medical records | Smoking cessation | Adding tobacco status as a vital sign resulted in an increase in some clinical guideline recommended actions, particularly documentation of smoking status | While the use of electronic medical records to prompt or provide feedback on the clinical treatment of tobacco dependence demonstrates some positive results | 5 | **+** |
| Adaji 2008 | To demonstrate the benefits of information technology in supporting a systematic approach to diabetes management in general practice | Information technology | Patients' health outcomes | Information technology has been used to provide support to patients, enhance changes in healthcare delivery and provide clinicians with access to expertise and timely, useful data about individual patients and populations. Information technology use has been associated with a corresponding improvement in measures of diabetes care | Information technology can be used to improve diabetes care by promoting a productive and informative interaction between the patient and the care team | 5 | **-** |
| Calabretto 2005 | To identify electronic decision supported systems that directly support pharmacists or pharmacy practice, in either the hospital or community settings | Elecronic decision support system | Pharmacist practice | Three studies described alerting systems for pharmacists, and one described the effect on pharmacist workflow of computerised prescribing | There is scant literature describing electronic decision support system activities specifically for pharmacy or pharmacists, in comparison to the substantial quantity of similar literature for healthcare in general | 5 | **-** |
| Curtain 2014 | To determine whether CDSS in community pharmacy practice can improve medication use and patient outcomes | CDSSs | Medication use and patient outcomes | Six studies showed statistically significant improvements in the measured outcomes: increased patient counselling, 31% reduced frequency of drug–drug interactions, reduced frequency of inappropriate medications in the elderly and in pregnant women, and increased pharmacists’ interventions for under-prescribed low-dose aspirin and over-prescribed high-dose proton pump inhibitors | Most studies showed improved prescribing practice, via direct communication between pharmacists and doctors or indirectly via patient education | 1 | **+** |
| **Environmental restructuring** *(Changing the physical or social context)* | | | | | | | |
| Kuethe 2013 | To review the effectiveness of nurse-led asthma care provided | Nurse-led care | Frequency of exacerbations, Asthma severity, and symptoms, health care costs | No significant difference between nurse-led care for patients with asthma compared to physician-led care for the outcomes assessed | Nurse-led care may be appropriate in patients with well-controlled asthma | 11 | **+** |
| Legare 2010 | To determine the effectiveness of interventions to improve healthcare professionals’ adoption of shared decision making | Shared decision making | Healthcare professionals’ adoption of shared decision making | Interventions targeting both patients and healthcare professionals had a positive effect compared to usual care (SMD: 2.83) and compared to interventions targeting patients alone (SMD: 1.42) | It is uncertain whether interventions to improve adoption of shared decision making are effective given the low quality of the evidence | 11 | **-** |
| Unverzagt 2014 | To assess implementation strategy which can improve physician adherence to the recommendations of guidelines in primary care | Multiple interventions | Physicians adherence rate to guidelines | The strongest benefit was noted with organizational change (odds ratio 1.96; 95% CI 1.4 to 2.75), followed by provider education and provider reminder systems | Major strategies should be well considered in guideline implementation: organizational changes in the primary care team and provider education | 10 | **+** |
| Smith 2007 | To determine the effectiveness of shared-care health service interventions designed to improve the management of chronic disease across the primary-specialty care interface | Shared-care interventions | Physical health outcomes, mental health outcomes, and psychosocial health outcomes, treatment satisfaction, measures of care delivery | Overall no consistent improvements in physical or mental health outcomes, psychosocial outcomes, psychosocial measures including measures of disability and functioning, hospital admissions, default or participation rates, recording of risk factors and satisfaction with treatment | No evidence to support the widespread introduction of shared care services at present | 10 | **-** |
| Urquhart 2009 | To assess the effects of nursing record systems on nursing practice and patient outcomes | Nursing record system | Nursing practice and patient outcomes | Nursing care planning systems and total nurse record demonstrated uncertain or equivocal results | There is a limited evidence of effects on practice attributable to changes in record systems | 10 | **-** |
| Laurant 2005 | To evaluate the impact of doctor-nurse substitution in primary care on patient outcomes, process of care, and resource utilisation including cost | Nurse-led care | Patient outcomes, process of care, and resource utilisation including cost | No appreciable differences were found between doctors and nurses in health outcomes for patients, process of care, resource utilisation or cost | The findings suggest that appropriately trained nurses can produce as high-quality care as primary care doctors and achieve as good health outcomes for patients | 10 | **+** |
| Proia 2014 | To assess the effectiveness of team-based care in improving blood pressure outcomes | Team based care | Patients with controlled BP, reduction in systolic or diastolic BP | 12% of patients with improvement in BP | Team-based care increased the proportion of people with controlled blood pressure | 9 | **+** |
| Page 2005 | To evaluate the best available evidence related to nurse-led cardiac clinics | Any interventions in nurese-led care | Adverse event rates, re-admisions, clinical and cost-effectiveness, consumer satisfaction and compliance with therapy | Although not all outcomes obtained statistical significance, nurse-led clinics were at least as effective as GPs clinics for most outcomes | Nurse-led clinics were at least as effective as GPs clinics for most outcomes | 9 | **-** |
| Damiani 2013 | To assess whether group practice has a more positive impact compared with the single-handed practice on different aspects of health care | Group versus single handed practice, information and communication technology | Clinical process measures, doctor's and patient's perspectives | A positive impact of group medicine on clinical process measures and throughput, doctor’s perspective, Innovation, information and communication technology and quality assurance was found. | Group practice might be a successful organizational requirement to improve the quality of clinical practice in primary health care | 8 | **-** |
| Health 2013 | To assess the effectiveness of specialized nurses in patient care to optimize chronic disease management among adults | Task shifting | Health outcomes | No significant differences in health resource use, disease-specific measures, quality of life, or patient satisfaction. A reduction in hospitalizations and improved management of BP and lipids among patients with coronary artery disease | Specialized nurses with an autonomous role in patient care had comparable outcomes to physicians alone, with consistent results among a subgroup analysis of patients with diabetes based on low-quality evidence | 8 | **+** |
| Desborough 2012 | To evaluate the efficacy of nurse-led primary healthcare walk-in centers | Nurse-led care | Users, quality of care provided, impact on other health care providers, internal and external perceptions, satisfaction | The impact is not clearly presented. | Uncertain. The possibility that walk-in centers create demand highlights the need for clearer evidence of the drivers of demand for health care in walk-in centers | 8 | **-** |
| Schadewaldt 2011 | To assess effective interventions in nurse-led cardiac clinics including patient education, risk factor assessment and continuity of care | Multiple interventions | Level of care and health outcomes in cardiac patients | The major nurse-led intervention applied in the clinics consisted of health education, counselling behaviour change, and promotion of a healthy lifestyle. No firm outcome was derived | Care was equivalent to non-nurse-managed clinics, and there was no greater risk of poorer outcomes in the nurse-led clinics | 8 | **-** |
| Mitchell 2008 | To assess the impact of coordinated multidisciplinary care in primary care, represented by the delivery of formal care planning by primary care teams or shared across primary-secondary teams, on outcomes in stroke, relative to usual care | Multi-disciplinary primary care team | Outcomes in stroke | Mortality rates are not impacted by multidisciplinary care planning | While multidisciplinary care planning may not unequivocally improve the care of patients with completed stroke, there may be process benefits such as improved task allocation between providers | 8 | **-** |
| Gilbody 2008 | To establish the effectiveness of screening in improving the recognition of depression, the management of depression, and the outcomes of patients with depression | Screening and case-finding instruments | Improving the recognition of depression, the management of depression, and the outcomes of patients with depression | The use of screening or case-finding instruments were associated with a modest increase in the recognition of depression by clinicians (RR 1.27, 95% CI 1.02 to 1.59), had no impact on recognition (RR 1.03, 95% CI 0.85 to 1.24) | If used alone, case-finding or screening questionnaires for depression appear to have little or no impact on the detection and management of depression by clinicians | 7 | **-** |
| Dennis 2009 | To assess the effectiveness of task substitution between GPs and pharmacists, GPs and nurses for the care of older people with chronic disease | Task shifting | Improved disease control | Task substitution between pharmacists and GPs and nurses and GPs resulted in an improved process of care and patient outcomes, such as improved disease control | Task substitution is important for the health professionals' roles and to encourage multidisciplinary teams | 6 | **+** |
| Courtenay 2008 | To identify, summarise and critically appraise the current evidence regarding the impact and effectiveness of nurse-led care in acute and chronic pain | Nurse-led care | Patients' outcomes | Educational interventions and the use of protocols by specialist nurses can improve patients understanding of their condition and improve pain control. Acute pain teams, led by nurses, can reduce pain intensity and are cost effective | Nurses play key roles in the diverse range of models of care that exist in acute and chronic pain. However, there are methodological weaknesses and under-researched issues that point to a need for further rigorous evaluation | 4 | **+** |
| Riley 2010 | To assess the effectiveness of group visits for persons with diabetes | Group visits | Health outcomes in diabetes patients | Group visits failed to demonstrate consistent statistical improvement in health outcomes | Group visits did not improve significant health outcomes | 3 | **-** |
| Carey 2007 | To identify systematically, summarize and critically appraise the current evidence regarding the activity and effects of nurse-led care in diabetes | Nurse-led care | Diabetes patient outcomes | Improved glycemic control, diabetic symptoms, cost-effectiveness and decreased length of hospital stay are the main benefits of nurse-led interventions in diabetes care | Findings of the review are generally positive | 3 | **+** |
| Martelly 2014 | To investigate the clinical effectiveness and costs of nurses working as substitutes for physicians in primary care | Nurse-led care | Nurse-led care appears to have a positive effect on patient care and outcomes | Nurse-led care was effective at reducing the overall risk of hospital admission (RR 0.76, 95% CI 0.64–0.91) and mortality (RR 0.89, 95% CI 0.84–0.96) | Nurse-led care appears to have a positive effect on patient care and outcomes | 0 | **+** |
| **Incentivisation** *(Creating expectation of reward)* | | | | | | | |
| Houle 2012 | To evaluate the effect of P4P remuneration targeting individual health care providers | P4P | Improved screening; quality of care for CDs | Uncontrolled studies suggested that P4P improves quality of care, but higher-quality studies with contemporaneous controls failed to confirm these findings | The effect of P4P targeting individual practitioners on quality of care and outcomes remains largely uncertain | 9 | **-** |
| Gillam 2012 | To evaluate impact of P4P on the quality of primary medical care | P4P | Quality of primary care | The quality of care for incentivized conditions during the first year of the framework improved at a faster rate than the preintervention trend and subsequently returned to prior rates of improvement. | Observed improvements in quality of care for chronic diseases were modest, and the impact on costs, professional behaviour, and patient experience remains uncertain. | 9 | **-** |
| Scott 2011 | To examine the effect of changes in the method and level of payment on the quality of care provided by PCPs | Financial incentives | Quality of care delivered by physicians | Six of the 7 studies showed positive but modest effects on quality of care for some primary outcome measures, but not all | There is insufficient evidence to support or not support the use of financial incentives to improve the quality of primary health care | 9 | **-** |
| Langdown 2014 | To assess the efficacy of the quality and outcomes framework as part of the pay-for-performance scheme for improving health outcomes | P4P | Health improvements | Findings provide strong evidence that the quality and outcomes framework initially improved health outcomes for a limited number of conditions but subsequently fell to the pre-existing trend | The quality and outcomes framework has limited impact on improving health outcomes due to its focus on process-based indicators and the indicators’ ceiling thresholds | 8 | **-** |
| Eijkenaar 2013 | A comprehensive overview of effects of P4P in a broad sense by synthesizing findings from published SRs | P4P | Cost effectiveness | Findings suggest that P4P can potentially be cost-effective, but the evidence is not convincing; many studies failed to find an effect and there are still few studies that convincingly disentangled the P4P effect from the effect of other improvement initiatives | Although data is available on a wide variety of effects, strong conclusions cannot be drawn due to a limited number of studies with strong designs | 7 | **-** |
| Vahidi 2013 | To discuss the impact of different methods of payment to FPs and general practitioners, quantity of service provision and referral rate behaviour | Payment mechanisms to FPs | Health system outcomes | In comparison to salary and capitation, fee-for-service (FFS) was associated with 9%-12% lower referral rate presumably because physicians wanted to treat patients and increase their incomes by producing more services | No firm conclusion was derived. | 5 | **-** |
| McDonald 2008 | To review the implementation and impact of different funding initiatives across the health systems | Funding initiatives or incentives | Health outcomes and satisfaction | In Australia, financial incentives have been the main mechanism for bringing about change, whereas, in both England and New Zealand, they are part of a broader range of funding reforms including the introduction of capitation and practice-based commissioning | Individual, patient-level, financial incentives may present significant impediments for population subgroups with complex needs | 3 | **-** |
| **Modelling** *(Providing an example for people to aspire to or imitate)* | | | | | | | |
| Flodgren 2011 | To assess the effectiveness of the use of local opinion leaders in improving professional practice and patient outcomes | Local opinion leaders | Measures of professional performance and/or health outcomes | The effects of interventions varied across the 63 outcomes from 15% decrease in compliance to 72% increase in compliance with desired practice | Opinion leaders alone or in combination with other interventions may successfully promote evidence-based practice, but effectiveness varies both within and between studies | 9 | **+** |
| Harkness 2009 | To assess the effects of on-site mental health workers delivering psychological therapy and psychosocial interventions in primary care on the clinical behaviour of PCPs | Mental health workers involvement | Consultation rates, prescribing, and referral | Mental health workers caused significant reductions in PCP consultations (SDM -0.17, 95% CI -0.30 to -0.05), psychotropic prescribing (RR 0.67, 95% CI 0.56 to 0.79), prescribing costs (SDM -0.22, 95% CI -0.38 to -0.07), and rates of mental health referral (RR 0.13, 95% CI 0.09 to 0.20) for the patients at primary care | Mental health workers working in primary care to deliver psychological therapy and psychosocial interventions cause a significant reduction in PCP behaviours such as consultations, prescribing, and referrals to specialist care | 10 | **+** |
| **Multiple interventions** | | | | | | | |
| Sandall 2013 | To compare midwife-led continuity models of care with other models of care for childbearing women and their infants | Mid-wife led continuity model | Differences in morbidity and mortality, effectiveness and psychosocial outcomes | Women who had midwife-led continuity models of care were less likely to experience regional analgesia (RR 0.83, 95% CI 0.76 to 0.90), episiotomy (RR 0.84, 95% CI 0.76 to 0.92), and instrumental birth (RR 0.88, 95% CI 0.81 to 0.96), and were more likely to experience no intrapartum analgesia/anaesthesia (RR 1.16, 95% CI 1.04 to 1.31), spontaneous vaginal birth (RR 1.05, 95% CI 1.03 to 1.08), attendance at birth by a known midwife (RR 7.83, 95% CI 4.15 to 14.80), and a longer mean length of labour (MD 0.50, 95% CI 0.27 to 0.74) | Most women should be offered midwife-led continuity models of care and women should be encouraged to ask for this option although caution should be exercised in applying this advice to women with substantial medical or obstetric complications | 11 | **+** |
| Dwamena 2012 | To assess the effects of interventions for healthcare providers that aim to promote patient-centered care approaches in clinical consultations | Any interventions | Shared treatment decision-making as a marker of patient-centered care | Studies using complex interventions that focused on providers and patients with condition-specific materials generally showed benefit in health behaviour and satisfaction, as well as consultation processes, with mixed effects on health status | Interventions to promote patient-centerd care within clinical consultations are effective across studies in transferring patient-centerd skills to providers. However, the effects on patient satisfaction, health behaviour, and health status are mixed | 11 | **-** |
| Thota 2012 | To evaluate the effectiveness of collaborative care models to improve the management of depressive disorders | Collaborative care models | Depression disorder management improvement markers | Robust evidence of effectiveness of collaborative care in improving depression symptoms (SMD 0.34); adherence to treatment (OR 2.22); response to treatment (OR 1.78); remission of symptoms (OR 1.74); recovery from symptoms (OR 1.75); quality of life/functional status (SMD 0.12); and satisfaction with care (SMD 0.39) for patients diagnosed with depression | Collaborative care models are effective in achieving clinically meaningful improvements in depression outcomes and public health benefıts in a wide range of populations, settings, and organizations, especially at the primary care level | 11 | **+** |
| McKinstry 2006 | To assess the effects of interventions intended to improve patients’ trust in doctors or a group of doctors | Informative, educational, multiple interventions | Trust | A small but statistically-significant increase in trust | There remains insufficient evidence to conclude that any intervention may increase or decrease trust in doctors | 11 | **-** |
| Rolfe 2014 | To assess the effects of interventions intended to improve patients’ trust in doctors or a group of doctors | Interventions (informative, educational, behavioural, organisational) | Trust level | Trials showing a small statistically significant increase in trust included interventions of physician disclosure of financial incentives; providing a choice of physician based on concordance between patient and physician beliefs about care; group visits | Insufficient evidence to conclude that any intervention may increase or decrease trust in doctors | 10 | **-** |
| Jackson 2013 | To assess effects of patient centered medical home on patient and staff experiences, process of care, and clinical and economic outcomes | PCMH | Emergency department visits; hospital admissions | Staff experiences were improved by a small to a moderate degree. Evidence suggested a reduction in emergency department visits but not in hospital admissions in older adults | The patient centered medical home holds promise for improving the experiences of patients and staff and potentially for improving care processes | 10 | **+** |
| Baishnab 2012 | To determine the effectiveness of organised asthma care delivered via primary care based asthma clinics | Organised asthma care | Hospitalization due to asthma exacerbations, accident and emergency visit, use of reliever and preventer medication, and quality of life | No statistically significant difference between the asthma clinic group and the control group for most outcomes | There is limited evidence of efficacy for primary care based asthma clinics, and firm conclusions cannot be formed until more good quality trials have been carried out | 10 | **-** |
| Lau 2012 | To assess effectiveness of QI interventions for increasing the vaccination rates among community-dwelling adults | QI | Improved vaccination rate | Interventions were associated with improvements in the rates of any vaccination (111 comparisons in 77 studies, pooled odds ratio = 1.61, 95% CI, 1.49-1.75) | QI interventions can modestly improve vaccination rates in community-dwelling adults | 10 | **+** |
| Archer 2012 | To assess the effectiveness of collaborative care for patients with depression or anxiety | Colloborative care | Improvement in depression and anxiety outcomes, medication use, mental health quality of life, and patient satisfaction | The results of primary analyses demonstrated significantly greater improvement in depression outcomes for adults with depression treated with the collaborative care model in the short-term (SMD -0.34, 95% CI -0.41 to -0.27; RR 1.32, 95% CI 1.22 to 1.43), medium-term (SMD -0.28, 95% CI -0.41 to -0.15; RR 1.31, 95% CI 1.17 to 1.48), and long-term (SMD -0.35, 95% CI -0.46 to - 0.24; RR 1.29, 95% CI 1.18 to 1.41) | Collaborative care is associated with significant improvement in depression and anxiety outcomes compared with usual care and represents a useful addition to clinical pathways for adult patients with depression and anxiety | 10 | **+** |
| Laliberte 2011 | To evaluate the effectiveness of primary care interventions to improve the detection and treatment of osteoporosis | Multiple interventions | Incidence of bone mass density testing; osteoporosis treatment initiation | Pooling the results of six trials showed an increased incidence of osteoporosis treatment initiation (RD: 20%; 95% CI: 7–33%) and of bone mineral density testing and/or osteoporosis treatment initiation (RD: 40%; 95% CI: 32–48%) for high-risk patients following intervention | Multifaceted interventions targeting high-risk patients and their primary care providers may improve the management of osteoporosis, but improvements are often clinically modest | 10 | **+** |
| Akbari 2008 | To estimate the effectiveness and efficiency of interventions to change outpatient referral rates or improve outpatient referral appropriateness | Multiple interventions | Provider performance or health outcomes | Effective strategies included dissemination of guidelines with structured referral sheets (4 of 5 studies) and involvement of consultants in educational activities (2 of 3 studies). Organisational interventions were effective | A limited number of rigorous evaluations to base policy on. Active local educational interventions involving secondary care specialists and structured referral sheets are the only interventions shown to impact on referral rates | 10 | **-** |
| Arnold 2005 | To assess the effectiveness of professional interventions, alone or in combination, in improving the selection, dose and treatment duration of antibiotics prescribed by healthcare providers in the outpatient setting | Any interventions | Reducing the incidence of antimicrobial resistant pathogens | Interactive educational meetings appeared to be more effective than didactic lectures. Educational outreach visits and physician reminders produced mixed results | No single intervention can be recommended for all behaviours in any setting. Multi-faceted interventions where educational interventions occur on many levels may be successfully applied to communities after addressing local barriers to change | 10 | **+** |
| Majka 2014 | To evaluate care coordination and/or team approach methods in the management of patients requiring long-term enteral tube feeding | Care coordination and/or team approach methods; multiple simultaneous strategies | Reductions in complications; hospitalization rate | No significant reductions in complications, infections, and overall hospital admissions, but significant reduction of total hospital costs after the interventions | A positive association of care coordination by a multidisciplinary team approach and improved patient outcomes for long-term enteral feeding patients was noted | 9 | **-** |
| De Belvis 2009 | To assess the effectiveness of evidence-based medicine tools available to PCPs to improve the quality of T2DM disease management | Evidence based medicine tools | Improvement in the quality of T2DM disease management | As little evidence that adherence of primary care professionals to evidence-based medicine tools improves both outcomes and process indicators of diabetes management | The adherence to evidence-based medicine instruments is likely to improve process of care, rather than patient outcomes | 9 | **-** |
| Okelo 2013 | To assess the effect of interventions designed to improve health care providers’ adherence to asthma guidelines on health care process outcomes, clinical outcomes, health care processes | Any interventions | Health cares process outcomes, clinical outcomes, health care processes | There was moderate evidence for increased prescriptions of asthma controller medications using decision support, feedback, and audit, and clinical pharmacy support interventions and low-grade evidence for organizational change, multicomponent interventions | There is low to moderate evidence to support the use of decision support tools, feedback, and audit, and clinical pharmacy support to improve the adherence of health care providers to asthma guidelines, as measured through health care process outcomes, and to  improve clinical outcomes | 8 | **+** |
| Mansell 2011 | To identify interventions that reduce primary care delay in the referral of patients with cancer to secondary care | Multiple interventions | Reduction in delay in cancer referral | Education, audit and feedback, decision support software and guideline use, diagnostic tools, and other specific skills training were identified. No study measured a direct effect on reducing delay | There was no evidence that any intervention directly reduced primary care delay in the diagnosis of cancer | 8 | **-** |
| Guy 2011 | To assess the efficacy of interventions for increasing the uptake of chlamydia screening in primary care | Multiple interventions | Chlamydia screening rate | Of the 15 interventions among females, six were associated with significant increases in screening rates. Of the six interventions targeting males, two found significant increases in screening rate | Interventions that promoted the universal offer of a chlamydia test in young people had the greatest impact on increasing screening in primary care | 8 | **-** |
| Gallagher 2010 | To assess the effectiveness of QI interventions to reduce systolic BP in people with chronic kidney disease in primary care, in order to reduce cardiovascular risk and slow the progression of renal disease | QI strategies | Change or reduction in BP and other hypertension markers | Three RCTs showed a combined effect of a reduction in systolic BP of 10.50 mmHg (95% CI = 5.34 to 18.41 mmHg). One non-RCT showed a reduction in systolic BP of 9.30 mmHg (95% CI = 3.01 to 15.58 mmHg) | Quality improvement interventions can be effective in lowing blood pressure, and potentially in reducing cardiovascular risk and slowing progression in chronic kidney disease | 8 | **+** |
| Ranji 2008 | To assess the effectiveness of QI strategies to reduce antibiotic prescribing for acute outpatient illnesses for which antibiotics are often inappropriately prescribed | QI strategies | The absolute reduction in the proportion of patients receiving antibiotics. | The median reduction in the proportion of subjects receiving antibiotics was 9.7% interquartile range (IQR), 6.6–13.7% over 6 months median follow-up. | QI efforts are effective at reducing antibiotic use in ambulatory settings, although much room for improvement remains | 8 | **+** |
| Fahey 2005 | To determine the effectiveness of educational and organisational strategies used to improve control of BP | Educational and organisational strategies | BP control measures | An organised system of regular review allied to vigorous antihypertensive drug therapy was shown to reduce BP and all-cause mortality at 5 years follow-up (6.4% versus 7.8%, difference 1.4%) in a single large RCT. Educational interventions directed towards physicians were associated with small reductions in systolic BP | General practices and community-based clinics need to have an organised system of regular follow-up and review of their hypertensive patients | 8 | **+** |
| Moe-Byrne 2014 | To summarise evidence on the effectiveness of behaviour change interventions to encourage prescribing of generic forms of prescription drugs | Any interventions | Improvement in generic drugs prescriptions | Educational intervention, collaboration with pharmacists, electronic prescribing and multifaceted interventions improved generic prescribing | Financial incentives with educational intervention and audit/feedback found effective but decision-makers should take into account the practicality and costs of the interventions before implementation | 7 | **+** |
| McMillan 2013 | To assess efficacy of patient-centered care interventions for people with chronic conditions | Any interventions | Patient satisfaction, quality of care, health outcomes | It was difficult to draw firm conclusions because of the moderate to high risk of bias of the research designs | No firm conclusion | 7 | **-** |
| Newhouse 2011 | Compared to other providers (physicians or teams without advance practice nurse care) are advanced practice nurse care patient outcomes of care similar? | Advanced Practice Nurse care | Patient outcomes of care | The results indicate advanced practice registered nurses provide effective and high-quality patient care, have an important role in improving the quality of patient care | Advanced practice registered nurses provide safe, effective, quality care to a number of specific populations in a variety of settings | 7 | **+** |
| Saxena 2007 | To review the effectiveness of primary care interventions on glycemic control and cardiovascular risk factors in minority ethnic groups with diabetes | Case management | Clinical outcomes for diabetes in minority ethnic groups | Two main models of care were identified: (1) case management, with specialist diabetes nurses and community health workers and (2) the use of the services of link workers from minority ethnic groups to guide people with diabetes. Case management improved glycemic control (reduction in HbA1c range −0.5% to −1.75%) | Case management improves glycemic control and cardiovascular risk factors and link workers improve cardiovascular risk factor control | 7 | **+** |
| Grindrod 2006 | To determine which interventions are effective in influencing health practitioners’ prescribing practices and explore differences in intervention complexity, setting, sustainability, cost-effectiveness, and impact on patient outcomes | Any interventions | Patient outcomes | Consistently effective interventions included reminders, audit and feedback, educational outreach visits, organizational strategies, and patient-mediated interventions. Multi-faceted interventions were consistently shown to be more efficacious than single interventions | Interventions that are most effective for impacting prescribing practice include audit and feedback, reminders, educational outreach visits, and patient-mediated interventions | 7 | **+** |
| Shojania 2006 | To assess the impact on glycemic control of 11 distinct strategies for QI in adults with T2DM | QI strategies | Clinician behaviour or organizational change and reported changes in glycosylated hemoglobin (HbA1c) values | Interventions reduced HbA1c values by a mean of 0.42% (95% CI, 0.29%-0.54%) over a median of 13 months of follow-up | Most QI strategies produced small to modest improvements in glycemic control. Team changes and case management showed more robust improvements | 7 | **+** |
| Loganathan 2011 | To review systematically the effects of interventions to optimise prescribing in care homes | Any interventions | Improving appropriate prescribing or reducing appropriate prescribing inappropriate prescribing | Four intervention strategies were identified: staff education, multi-disciplinary team meetings, pharmacist medication reviews, and computerized decision support systems | Results are mixed and there is no one interventional strategy that has proved to be effective | 6 | **-** |
| Kaur 2009 | To identify interventions and strategies that can significantly reduce inappropriate prescribing in the elderly | Any interventions | Inappropriate prescribing | Interventions including computerized support system, pharmacist interventions, multidisciplinary case conferences found effective | Different strategies may be useful in reducing inappropriate prescribing in the elderly | 6 | **-** |
| Castelino 2009 | To review the impact of interventions by pharmacists on suboptimal prescribing in the elderly | Interventions for prescribing | Improving prescribing in the elderly | Most of the studies involving pharmacists showed significant improvement in suboptimal prescribing at one or more time points | Pharmacy services to reduce suboptimal prescribing found effective and noteworthy improvements | 6 | **+** |
| Christensen 2008 | To determine the effective components of depression care in primary care through a systematic examination of both general practice and community-based intervention trials | Community models of care | Improvement on the key depression measures | Components which significantly predict improvement were: revision of professional roles, the provision of a case manager and delivered a psychological therapy, and an intervention that incorporated patient preferences into care. Nurse, psychologist, and psychiatrist delivered care were effective, but pharmacist delivery was not | Case management is important in the provision of care in general practice. Certain community models of care (education programs) have potential while others are not successful in their current form (pharmacist monitoring) | 6 | **+** |
| Beach 2006 | To synthesize the findings of controlled studies evaluating interventions targeted at health care providers to improve health care quality or reduce disparities in care for racial/ethnic minorities | Provider and organization interventions | Health care quality | 10 studies that used a provider reminder system for provision of standardized services reported favorable outcomes | There are several strategies that may improve health care quality for racial/ ethnic minorities | 6 | **+** |
| Tory 2015 | To report and analyze QI efforts that are aimed at increasing adherence to preventive guidelines for glucocorticoid-induced osteoporosis. | QI measures | Glucocorticoid-induced osteoporosis prevention | Two studies of system changes showed significant improvements in glucocorticoid-induced osteoporosis prevention | None of the interventions produced robust changes, with overall adherence to glucocorticoid-induced osteoporosis guidelines remaining low. System-based interventions appeared more effective than education-based interventions | 5 | **-** |
| Huijg 2014 | To review literature on factors influencing PCPSs physical activity promotion practices | Any interventions | PHC professionals’ physical activity promotion behaviours | Many factors related to the development, delivery, and effects of the innovation, the sociopolitical and organizational culture, resources, and support, patient and PHC professional characteristics, and innovation strategies were identified as potential influences on PCPS promotion practices | This extensive overview of potential factors can inform intervention developers and implementers on which factors may play a role when introducing physical activity interventions in PHC | 5 | **-** |
| Zou 2012 | To identify clinic-based strategies for increasing screening and detection of bacterial sexually transmitted disease among gay men who have sex with men (MSM) | Any interventions | Sexually transmitted disease screening, rescreening, or detection rates | A significant increase in screening rates for using different strategies including use of a computer alert on an electronic medical record; the introduction of clinic guidelines on sexually transmitted disease screening; and short text messaging reminders for repeat sexually transmitted disease screening, use of a computer alert on an electronic medical record, and an electronic medical record system | A range of interventions has been used, including the application of newer technologies targeting clinicians and patients that appear to be efficacious at increasing screening of MSM for the bacterial sexually transmitted disease | 5 | **+** |
| Jacobson 2011 | To evaluate interventions aimed at improving primary care providers’ identification, assessment, prevention and/or management of obesity in children and adolescents | Multiple interventions | Weight lose and behaviour change | The interventions most frequently utilized the elements of self management support (69%), decision support (100%), delivery system support (77%) and clinical information systems (23%) | Intervention programmes that included more components of the chronic care model were more effective | 5 | **+** |
| Smit 2007 | To review the strategies used for improvement of routine treatment in terms of their effects on patient outcome | Psychological and supportive interventions | Patient outcomes | Three strategies: (1) training primary care physicians – this appears ineffective (2) supporting PCPs by other professionals – this produces better short-term outcomes but does not prevent recurrence (3) organisational quality improvement – this shows improved outcomes at 6 months, and there is some evidence of longer term effectiveness | Effects of the reviewed strategies generally do not seem to persist over time and no clear superiority over usual care has been demonstrated | 5 | **-** |
| Van Cleave 2012 | To review practice-based interventions to increase the proportion of patients receiving recommended screening and follow-up services in pediatric primary care | QI initiatives, electronic records | Screening and follow-up rate | Interventions were heterogeneous and often multifaceted, and several types of interventions, such as provider/staff training, electronic medical record templates/prompts, and learning collaboratives, appeared effective in improving screening quality | Several feasible, practice- and provider-level interventions appear to increase the quality of screening in pediatric primary care | 4 | **+** |
| Gask 2011 | To review educational intervention for medically unexplained symptoms in primary care settings | Reattribution model | Physician level: acquisition of skills, diagnostic behaviour, attitudes; Patient outcomes | Impact on clinical outcomes has been mixed | No firm conclusion | 4 | **-** |
| Phillips 2010 | To review different models of clinical governance and to explore their relevance to Australian primary health care, and their potential contributions on quality and safety | Different models using various interventions | Health outcomes, governance level | Most evidence supports governance models which use targeted, peer-led feedback on the clinician's own practice | Locally relevant clinical indicators, the use of computerised medical record systems, regional primary health care organisations that have the capacity to support the uptake of clinical governance at the practice level, and learning from the Aboriginal community controlled sector will help integrate clinical governance into primary care | 4 | **+** |
| Dennis 2008 | To review the effectiveness of chronic disease management interventions for physical health problems in the primary care setting, and to identify policy options for implementing successful interventions in Australian primary care | Any interventions | Health professional performance and patient-level measures of disease control, patient satisfaction | The interventions most likely to be effective were engaging primary care in self-management support through education and training for general practitioners and practice nurses, and including self-management support in care plans linked to multidisciplinary team support | The chronic care model provides a useful framework for understanding the impact of chronic disease management interventions and highlights the gaps in evidence | 3 | **+** |
| Gunten 2007 | To identify and review the clinical and economic impact of pharmacists’ interventions on antibiotic use | Pharmacists’ interventions | Antimicrobial therapy at patient’s or prescriber’s level | Interventions were often combined to provide a multifaceted intervention, making it difficult to isolate the impact of one specific intervention | The most frequently observed outcomes with a positive impact were appropriateness of prescribing and cost savings | 2 | **-** |
| **Persuasion** *(Using communication to induce positive or negative feelings or stimulate action)* | | | | | | | |
| Jenkins 2015 | To assess the effectiveness of interventions to reduce the use of imaging for low-back pain | Audit-feedback, reminders, clinical decision support on imaging | Reduced referrals for imaging | Targeted reminders to primary care physicians of appropriate indications for imaging reduced referrals for imaging by 22.5% (95% CI 8.4% to 36.8%). Interventions including audits and feedback, education or guideline dissemination did not significantly reduce imaging rates | Targeted reminders to primary care doctors were effective interventions in reducing the use of imaging for low-back pain | 10 | **+** |
| Siddiqui 2011 | To evaluate the role of physician reminders in faecal occult blood testing for colorectal cancer screening | Reminders | Screening rate | A higher percentage uptake was noted when physician reminders were given. However, only 2 studies found the percentage uptake significantly higher | Reminding physicians about those patients due for testing may not improve the effectiveness of a colorectal cancer screening programme | 10 | **-** |
| Holt 2012 | To assess the influence on clinical behaviour of patient-specific electronically generated reminders available at the time of the clinical encounter | Reminder | Rates of screening, vaccination, diagnostic tests, BP measurement, BP control, rate of venous thromboembolism, and measures of prescribing quality | An overall OR of 1.79 (95% CI 1.56, 2.05) in favour of reminders was derived | A moderate effect of electronically generated, individually tailored reminders on clinician behaviour during the clinical encounter | 9 | **+** |
| Lu 2008 | To update systematic review of interventions to improve the quality and efficiency of medication use in the US managed care setting | Any interventions | Medication use, process and patient outcomes | Effective interventions included one-to-one academic detailing, computerized alerts and reminders, pharmacist-led collaborative care, and multifaceted disease management | Computerized alerts showed promise in improving short-term outcomes but little is known about longer-term outcomes | 6 | **+** |
| **Training** *(Imparting skills)* | | | | | | | |
| Horvat 2014 | To assess the effects of cultural competence education interventions for health professionals on patient-related outcomes, health professional outcomes, and healthcare organisation outcomes | Cultural competence training | Health outcomes of patients | Two trials comparing cultural competence training with no training found no evidence of effect for treatment outcomes, including the proportion of patients with diabetes achieving LDL cholesterol control targets (RD -0.02, 95% CI -0.06 to 0.02), or change in weight loss (SMD 0.07, 95% CI - 0.41 to 0.55) | There was positive, albeit low-quality evidence, showing improvements in the involvement of culturally and linguistically diverse patients | 10 | **+** |
| Moore 2013 | To assess whether communication skill training is effective in improving the communication skills of healthcare professionals involved in cancer care, and in improving patient health status and satisfaction | Communication skills training | Patient health status and satisfaction | No significant differences between the groups with regard to outcomes assessing health care professionals ’burnout’, patient satisfaction or patient perception of the health care professionals communication skills was noted | Various communication skill training courses appear to be effective in improving some types of health care professionals communication skills related to information gathering and supportive skills | 10 | **+** |
| Sikorski 2012 | Does GP training in depression care affect patient outcome? | Training | Depression care | Training of providers alone did not result in improved patient outcomes. The additional implementation of guidelines and the use of more complex interventions in primary care yield a significant reduction in depressive symptomatology | Provider training by itself does not seem to improve depression care; however, if combined with additional guidelines implementation, results found effective for new-onset depression patient samples | 10 | **+** |
| Paskins 2014 | To identify the strengths, weaknesses and role of video stimulated recall | Video stimulated recall | GPs and patients relationships | Video-stimulated recall was identified as an important tool particularly add value to doctor’s post consultation accounts | Video-stimulated recall is particularly useful for study of specific consultation events | 7 | **+** |
| Eggenberger 2013 | To identify existent interventions to enhance communication in dementia care in various care settings | Communication skills training, education | Behaviour changes, changes in knowledge, skills, and attitude | Didactic methods included lectures, hands-on training, group discussions, and role-play | Communication skills training in dementia care significantly improves the quality of life and wellbeing of people with dementia and increases positive interactions in various care settings | 7 | **+** |
| Xu 2012 | To explore the use of simulated-patient methods in community pharmacy for non-prescription medicines | Simulated-patient methods | Counselling behaviour of pharmacy staff | The majority used simulated-patient methods to purely assess counselling behaviour of pharmacy staff, rather than as an opportunity to provide educational feedback to improve counselling behaviour | Few simulated-patient studies have incorporated performance feedback to encourage behaviour change and improve counselling skills | 7 | **-** |
| Soderlund 2011 | To evaluate different aspects of motivational training training for general health care professionals | Motivational interviewing training | Patient health outcomes | The training generated positive outcomes overall and had a significant effect on many aspects of the participants’ daily practice, but the results must be interpreted with caution due to the inconsistent study quality | Motivational training can be used to improve client communication and counselling concerning lifestyle-related issues in general health care | 7 | **+** |
| Lie 2011 | To assess the effects of cultural competency training on patient-centered outcomes | Cultural competency training | Patient outcomes improvements | Three studies reported positive (beneficial) effects; none demonstrated a negative (harmful) effect | There is limited research showing a positive relationship between cultural competency training and improved patient outcomes, but there remains a paucity of high-quality research | 6 | **-** |
| Henderson 2011 | To assess the effectiveness of culturally appropriate interventions to manage or prevent chronic disease in culturally and linguistically diverse communities | Cultural competency training | Changes in consumer health behaviour, utilisation ⁄ satisfaction with the service, and the cultural competence of healthcare providers | For CALD people with chronic disease, five intervention categories were identified: (1) the use of community-based bilingual health workers; (2) providing cultural competency training for health workers; (3) using interpreter service; (4) using multimedia and culturally sensitive videos, and (5) establishing community point-of-care services | The review supported the use of trained bilingual health workers, who are culturally competent, as a major consideration in the development of an appropriate health service model for culturally and linguistically diverse communities | 6 | **+** |
| Rashid 2010 | Benefits and limitations of nurses taking on aspects of the clinical role of doctors in primary care | Nurse training | Patient health outcomes | Impact in terms of practice change or patient health improvement is presented | There have been few studies in this key area of healthcare policy | 6 | **-** |
| Mesquita 2010 | To review the use of simulated patient methods to enhance communication skills of pharmacists | Simulated patient methods | Communication skills | The majority of studies had an assessment focus aimed at documenting counseling behaviour of practicing pharmacists, rather than an educational focus aimed at equipping pharmacists with effective communication skills | The majority of studies failed to describe the competencies and skills being investigated in relation to communication in the practice of pharmacy | 3 | **-** |
| **POLICY**  **Service provision** *(Delivering a service)* | | | | | | | |
| Wilson 2006 | To assess the effectiveness and efficiency of interventions to alter the length of primary care physicians’ consultations | Consultation time | Length of consultation | Altering appointment length resulted in modest changes in the average length of consultation. None of the interventions were associated with differences in patient satisfaction | The findings do not provide sufficient evidence to support or resist a policy of altering the lengths of primary care physicians’ consultations | 10 | **-** |
| Bhanbhro 2011 | To assess the non-medical prescribing in primary care | Non medical prescribing | Health care quality | Most studies reported that non-medical prescribing was widely accepted and viewed positively by patients and professionals | Paucity of studies; The gradual growth over time of legislative authority and in the numbers of non-medical prescribers, particularly nurses, in some countries suggests its importance | 9 | **-** |
| OHTA 2012 | To examine the role of SCBC in family practice | Specialized community-based care | Health resource utilization, quality of life | SCBC improved moderately hospitalization, readmission, emergency department visits, and quality of life | Specialized community-based care effectively improves outcomes in patients with heart failure, COPD, and diabetes. The effectiveness of SCBC in family practice is unclear | 8 | **+** |
| Wilson 2006 | To assess the effectiveness and cost-effectiveness of interventions to alter primary care physicians’ consultation length | Any interventions altering consultation time | Change in length of consultation; patient's satisfaction | Altering appointment length resulted in modest changes in average consultation length. None of the interventions were associated with differences in patient satisfaction | There is not sufficient evidence to support or resist a policy of altering consultation lengths of primary care physicians | 7 | **-** |
| McNaughton 2009 | To assess existing, brief nonpharmacologic interventions that are available for primary care physicians with minimal training in psychotherapy to use in managing depression in adult patients | Brief nonpharmacologic interventions | Improvement of symptoms on a validated depression scale | Significant improvements on depression scales were found in 6 out of 8 studies (P<.05) using various brief interventions and formal control groups | Bibliotherapy, cognitive behavioural therapy-based websites, and cognitive behavioural therapy-based computer programs might be effective in assisting primary care physicians who have minimal training in psychotherapy in treating adult patients with depression | 3 | **+** |
| **Communications** *(Using print, electronic, telephonic or broadcast media)* | | | | | | | |
| Sawmynaden 2012 | To assess the effects of email for the provision of information on disease prevention and health promotion, compared to standard mail or usual care, on outcomes for healthcare professionals, patients and caregivers, and health services, including harms | Email communication | Patient or caregiver behaviours/actions | There was no difference between email and standard mail (odds ratio 0.93; 95% CI 0.69 to 1.24) | The evidence on the use of email for the provision of information on disease prevention and health promotion was weak and inadequate | 11 | **-** |
| Cant 2011 | To review dietitians’ correspondence practices with recipient GPs regarding nutrition interventions recommended to patients | Dietitians’ correspondence practices | Patients outcomes, patients dietary change | Dietitians often fail to provide GPs with formalised correspondence that describes dietitians’ nutrition interventions with patients | Dietitians need skill in drafting reports or letters useful for general practitioners by selecting content of value to doctors and using a suitable style | 5 | **-** |
| Jiwa 2014 | What is the evidence that patients benefit from sound communication between primary care practitioners and nephrologists? | Communications | Patient outcomes | Some evidence of a direct impact from limited or inadequate communication on patient outcomes were noted | There is some evidence that improving the quality of letters from specialists to primary care practitioners may benefit patient care | 4 | **-** |
| **Guidelines** (Creating documents that recommend or mandate practice) | | | | | | | |
| Clarke 2010 | To assess effectiveness of guidelines for referral for elective surgical assessment | Guidelines | Appropriateness of referral including clinical appropriateness, appropriateness of destination and of pre-referral management, GPs knowledge of referral appropriate | Four RCTs reported increases inappropriateness of pre-referral care (diagnostic investigations and treatment) | Guidelines for elective surgical referral can improve appropriateness of care by improving prereferral investigation and treatment, but there is no strong evidence in favor of other beneficial effects | 8 | **-** |
| Ramsaroop 2007 | To assess the effect of increased advance directive completion in the primary care setting | Advance Directive | Difference in completion rates of advance directive | Absolute differences in completion rates varied from a high of 44% (favors intervention) to a low of –2% (favors control). A moderate overall effect in favor of the intervention | The most successful interventions incorporated direct patient–healthcare professional interactions over multiple visits | 5 | **+** |

RCTs = Randomized clinical trails; FP = Family physician; CME = Continuing medical education; GP = General physician; PCPs = Primary care providers; RTIs = Respiratory tract infections; T2DM = Type 2 diabetes mellitus; BP = Blood pressure; SMD = Standardized mean difference; RD = Risk difference; RR = Risk ratio; COPD = Chronic obstructive pulmonary disease.
